# Red-Crowned Crane Optimization: A Novel Biomimetic Metaheuristic Algorithm for Engineering Applications

**DOI:** 10.3390/biomimetics10090565

**Published:** 2025-08-24

**Authors:** Jie Kang, Zhiyuan Ma

**Affiliations:** 1 School of Mechanical and Electrical Engineering, Sanjiang University, Nanjing 210012, China; 13401832256@163.com; 2 Nanjing Agricultural Robotics and Equipment Engineering Research Center, Nanjing 210012, China

**Keywords:** metaheuristic algorithm, red-crowned crane optimization, exploration, exploitation

## Abstract

This paper proposes a novel bio-inspired metaheuristic algorithm called the Red-crowned Crane Optimization (RCO) algorithm. This algorithm is developed by mathematically modeling four habits of red-crowned cranes: dispersing for foraging, gathering for roosting, dancing, and escaping from danger. The foraging strategy is used to search unknown areas to ensure the exploration ability, and the roosting behavior prompts cranes to approach better positions, thereby enhancing the exploitation performance. The crane dancing strategy further balances the local and global search capabilities of the algorithm. Additionally, the introduction of the escaping mechanism effectively reduces the possibility of the algorithm falling into local optima. The RCO algorithm is compared with eight popular optimization algorithms on a large number of benchmark functions. The results show that the RCO algorithm can find better solutions for 74% of the CEC-2005 test functions and 50% of the CEC-2022 test functions. This algorithm has a fast convergence speed and high search accuracy on most functions, and it can handle high-dimensional problems. The Wilcoxon signed-rank test results demonstrate the significant superiority of the RCO algorithm over other algorithms. In addition, applications to eight practical engineering problems further demonstrate its ability to find near-optimal solutions.

## 1. Introduction

Optimization algorithms are a class of computational methods used to find optimal or near-optimal solutions in complex problem spaces. Problems in many fields can be transformed into mathematical models that “maximize or minimize a certain objective function under specific constraints”, and optimization algorithms are the core tools for solving these problems. For example, in the field of engineering design, optimization algorithms can help explore design schemes with better performance, lower costs, or higher reliability [1,2]. In path planning problems, optimization can provide the path with the shortest distance or the least time to reach the destination [3,4]. For control systems, optimization methods can be used to determine controller parameters that enable the system to achieve better control performance [5,6]. In view of the demand for such technologies in real-world problems, many optimization methods have been proposed to solve these problems.

Optimization methods can generally be summarized as deterministic optimization methods and stochastic optimization methods. For some linear, low-dimensional, and continuous simple problems, traditional deterministic methods are sufficient to effectively solve them. However, most real-world problems exhibit complex characteristics such as nonlinearity, discreteness, and high dimensionality, which pose challenges for traditional methods to give satisfactory results. To address the challenges that are difficult to solve by deterministic optimization techniques, stochastic optimization techniques have emerged. Stochastic methods do not need to capture information from the objective function but exhibit random search behavior to provide suitable solutions to the problems. It should be noted that since these algorithms rely on the principle of random search, they often require multiple attempts before they can give a reliable solution.

Metaheuristic algorithms are a typical class of stochastic optimization methods for solving complex optimization problems [7,8]. They approach the optimal solution of a problem by utilizing candidate solutions in the search space and appropriate search strategies. The main characteristics of these algorithms include flexibility, robustness, and the ability to handle large-scale problems. Over the past few decades, metaheuristic algorithms have become one of the main methods for solving complex optimization problems and have been widely applied in many fields such as engineering optimization, energy management, power, and computer vision.

Metaheuristic algorithms are usually proposed by designers inspired by biological sciences, physical and chemical phenomena, and animal, plant, and human behaviors, which are used to solve real-world optimization problems. They can be roughly classified into four categories: evolution-based algorithms, swarm-based algorithms, human-based algorithms, and physics- and chemistry-based algorithms. The Genetic Algorithm (GA) [9] is the earliest evolutionary algorithm and was proposed in the 1970s. It searches for optimal solutions by simulating the processes of inheritance, crossover, and mutation in nature. Based on the idea of the GA, another representative Differential Evolution (DE) [10] was introduced in the 1990s. Swarm intelligence optimization algorithms acquire a near-optimal solution based on the wisdom of biological populations. These algorithms work through the synergistic behavior between individuals to complete tasks that individuals cannot complete independently. Particle Swarm Optimization (PSO) [11] is a very popular swarm intelligence algorithm originated from research on the foraging behavior of bird or fish populations. PSO has attracted widespread attention due to its advantages such as ease of implementation and few adjustment parameters. Since then, many new swarm intelligence algorithms have been proposed. Some examples are given below: the Artificial Bee Colony (ABC) [12] inspired by the cooperative foraging of bee colonies, the Fruit Fly Optimization Algorithm (FOA) [13], proposed based on the fact that fruit flies uses their keen senses of smell and vision for predation, the Grey Wolf Optimizer (GWO) [14], which is inspired by the hierarchy system and hunting behavior of gray wolves, the Butterfly Algorithm (BA) [15], which imitates the flight behavior of butterflies attracted by fragrance, the Whale Optimization Algorithm (WOA) [16], which simulates the hunting process of humpback whales, and the Dragonfly Algorithm (DA) [17], developed based on the foraging and migration behaviors of dragonflies. Human-based optimization algorithms are mainly inspired by daily human behaviors, such as management, cooperation, learning, and social behaviors. Several human-based algorithms are briefly described below. Rao et al. proposed Teaching–Learning-Based Optimization (TLBO) [18], which was inspired by the impact of teachers on student learning and interactive learning among students. The algorithm has been widely improved and applied due to its efficient optimization performance. Das et al. introduced Student Psychology-Based Optimization (SPBO) [19] by simulating the psychology of students expecting to improve their academic performance. In order to solve complex global optimization problems, Feng et al. proposed the Cooperation Search Algorithm (CSA) [20] inspired by the collaborative behavior of enterprise groups. Trojovská et al. developed the Chef-Based Optimization Algorithm (CBOA) [21] and Driving Training-Based Optimization (DTBO) [22] by simulating the process of chefs learning cooking skills and drivers learning driving skills, respectively. Physics- and chemistry-based algorithms mimic some of the physical and chemical phenomena in nature for the purpose of optimization search. Similarly, several examples are presented below, including the Gravitational Search Algorithm (GSA) [23] based on Newton’s law of gravity, the Multi-Verse Optimizer (MVO) [24] based on the theory of multi-verse evolution, the Material Generation Algorithm (MGA) [25] based on the chemical reactions of materials, and Fick’s Law Algorithm (FLA) [26] based on Fick’s first law of diffusion. In addition to the above four common classifications, there are other metaheuristic algorithms that are not included, such as Sine Cosine Algorithm (SCA) [27], Arithmetic Optimization Algorithm (AOA) [28], Hunger Games Search (HGS) [29], and Football-Game-Based Optimization (FGBO) [30]. Table 1 shows more metaheuristic algorithms and summarizes their inspirations. Note that since new optimization algorithms are constantly being proposed, it is impossible to cite all of them.

The core concepts of metaheuristic algorithms are exploration and exploitation [39]. In general, the design goal of all algorithms is to balance the exploration and exploitation phases so that the search agents efficiently converge to the near-optimal solutions [40]. Although various algorithms have been proposed, many of them are unsatisfactory when dealing with high-dimensional and multimodal problems. This is because their inappropriate search mechanisms make them susceptible to local optima. For example, the GWO [14], SCA [27], and Golden Jackal Optimization (GJO) algorithms [41] all require search individuals to move towards the optimal position found in the current iteration. This mechanism leads to a gradual decay in population diversity during the iteration process, which weakens the exploration capability of the algorithms, resulting in limited optimization performance in high-dimensional or multi-modal problems. For the HHO algorithm [33], it achieves the transition from global exploration to local exploitation through an energy factor (which decreases linearly with the number of iterations). However, this mode is highly dependent on the effectiveness of the exploration phase. If the algorithm fails to find high-quality candidate solutions during the exploration phase, it will easily fall into local optima during the exploitation phase. In addition, we find that some algorithms, such as the Slime Mould Algorithm (SMA) [42], lack effective strategies to jump out of local optima, thus performing poorly on multi-modal functions.

By studying the habits of red-crowned cranes, we have found that their behaviors such as foraging, roosting, and escaping are similar to the processes of exploration, exploitation, and jumping out of local optima in metaheuristic algorithms. Therefore, this study aims to develop an algorithm model capable of dealing with certain complex multimodal and high-dimensional problems based on the behaviors of red-crowned cranes. The main contributions of this work are as follows:

1. A biomimetic RCO algorithm is proposed, which simulates the four behaviors of red-crowned cranes in nature: dispersing for foraging, escaping from danger, gathering for roosting, and crane dance. The foraging strategy is used to search unknown areas to ensure the exploration ability, and the roosting behavior prompts cranes to approach better positions, thereby enhancing the exploitation performance. The crane dancing strategy further balances the local and global search capabilities of the algorithm. The introduction of the escaping mechanism effectively reduces the possibility of the algorithm falling into local optima.
2. The RCO algorithm is tested on CEC-2005 and CEC-2022 benchmark functions and is compared with eight popular algorithms from multiple perspectives, including optimization accuracy, convergence speed, rank-sum test, and scalability.
3. The RCO algorithm is used to optimize eight constrained application problems, and the ability of the RCO algorithm to deal with engineering design problems is compared with fifteen other optimization algorithms.

The framework of the rest of this paper is as follows: Section 2 describes the design inspiration of RCO and establishes its mathematical model. Section 3 gives the results and discussion of RCO on two types of test function sets. Section 4 tests the performance of RCO on engineering design problems. This work is summarized and the outlook for future research is indicated in Section 5.

## 2. Red-Crowned Crane Optimization (RCO)

### 2.1. Inspiration Source

**Figure 1:**
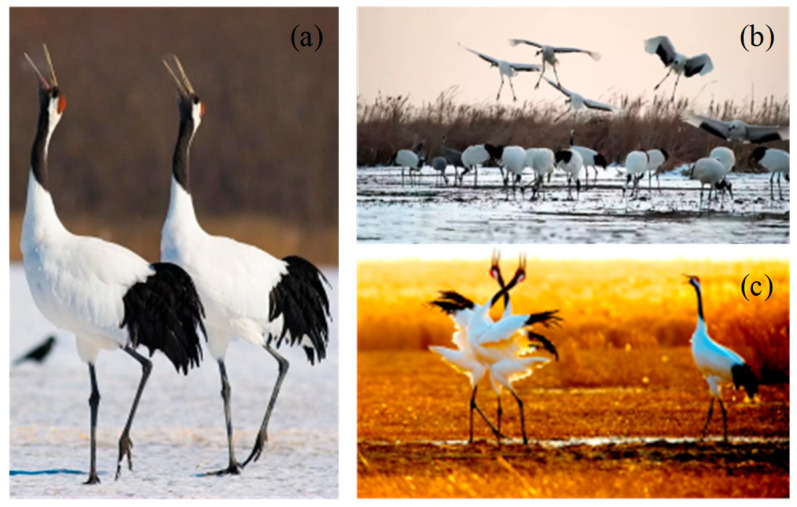
(**a**) Appearance of red-crowned cranes; (**b**) foraging behavior of red-crowned cranes; (**c**) dancing behavior of paired red-crowned cranes.

Red-crowned cranes are a type of large wading bird in the Gruidae family of the group Gruiformes, named after the red crowns on their heads. In general, they have a body height of 1.5 to 1.6 m, a body length of 1 to 1.5 m, a wingspan of 2.2 to 2.5 m, and a weight of 5 to 10.5 kg [43]. Male and female cranes are very similar in appearance, with their bodies almost pure white. As shown in Figure 1a, their heads are bare, featherless, and vermilion in color. The cheeks, throat and neck appear mostly dark brown. The primary flight feathers and the entire body feathers of the red-crowned cranes are all white, which is especially noticeable when they are flying [44]. The secondary and tertiary flight feathers appear black, and the tertiary flight feathers are long and curved, covering the tails. Therefore, the black feathers on their tails when they are standing are actually the tertiary flight feathers.

Red-crowned cranes symbolize happiness, longevity, and loyalty. Unfortunately, red-crowned cranes have become one of the rarest cranes in the world. At present, red-crowned cranes have been included in the IUCN Red List of Threatened Species and have also been included in the list of key protected wild animals in China [45,46]. In the world, red-crowned cranes are mainly distributed in China, Japan, Korea, Mongolia, and Russia. Red-crowned cranes in Japan usually do not migrate, while those in other countries migrate according to the seasons. Generally, red-crowned cranes leave their wintering grounds in February or March every year and go to areas such as the Russian Far East and Heilongjiang in China for breeding. In September or October, they migrate from their breeding grounds to Korea and east-central China for the winter [47].

During migration, several family groups usually gather into a larger group (up to 40–50 individuals, or even more than 100 individuals). However, after completing the migration, they still live in families or small groups. In addition, it has been observed that red-crowned cranes often forage in a certain area in pairs or alone, and sometimes in small groups. They eat a wide variety of foods, including fish, shrimp, tadpoles, clams, and the stems, leaves, and fruits of aquatic plants. Figure 1b shows red-crowned cranes foraging in shallow ponds. At night, red-crowned cranes roost in families in shoals or reed ponds surrounded by water. Whether they are foraging or resting, some adult red-crowned cranes are always particularly alert and constantly look up and around. Once they sense danger approaching, they take to the air and chirp loudly.

In late March or early April every year, red-crowned cranes begin their courtship behavior. During this period, the male crane turns its beak upward, raises its head and neck, looks up at the sky, spreads its wings, and sings loudly. The female crane responds loudly, and then they sing, jump, and dance with each other [48]. Their dance is very graceful, either stretching their necks and raising their heads, bending their knees and stooping, stepping in place, or jumping in the air, as shown in Figure 1c.

In this work, the foraging, escaping, roosting, and dancing behaviors of red-crowned cranes provide design inspiration and ideas for the RCO algorithm. These behaviors are analyzed and modeled in detail in the following section.

### 2.2. Population Initialization

The RCO algorithm proposed in this work is a new swarm-based metaheuristic algorithm that simulates the living habits of the red-crowned crane population. In the RCO algorithm, the positions of red-crowned cranes represent some candidate solutions for the problem to be solved. To find the near-optimal solution to this problem, it is necessary to continuously adjust the positions of the red-crowned cranes so that the candidate solutions are as close to the optimal solution as possible. Note that a sufficient number of candidate solutions (search agents) and adjustment steps (iterations) can increase the likelihood of finding the better solution. Therefore, like other swarm intelligence algorithms, the RCO algorithm initially needs to generate a random population *X_cranes_* in the search space, as follows:

(1) $$X_{cranes}=\left[\begin{array}{c} X_{1}\\ \vdots \\ X_{i}\\ \vdots \\ X_{n} \end{array}\right]=\left[\begin{array}{ccccc} x_{1,1} & \ldots & x_{1,j} & \ldots & x_{1,d}\\ \vdots & \ddots & \vdots & ⋰ & \vdots \\ x_{i,1} & \ldots & x_{i,j} & \ldots & x_{i,d}\\ \vdots & ⋰ & \vdots & \ddots & \vdots \\ x_{n,1} & \ldots & x_{n,j} & \ldots & x_{n,d} \end{array}\right],$$

(2) $$x_{i,j}=lb_{j}+r_{0}\cdot (ub_{j}-lb_{j}),i=1,2,\ldots ,n,j=1,2,\ldots ,d,$$

where *X_i_* is the position of the *i*-th red-crowned crane, *x_i_*_,*j*_ is the value of the *j*-th dimension (decision variable) of the *i*-th red-crowned crane, *n* is the number of red-crowned cranes, *d* is the number of decision variables, *r*_0_ is a random value in the range of 0 to 1, and *ub_j_* and *lb_j_* are the upper and lower bounds of the *j*-th decision variable, respectively.

In order to evaluate the quality of the candidate solutions, the fitness values of these candidate solutions for the objective function *F*(*X*) are calculated using Equation (3), and the solution corresponding to the best fitness value is recorded. As the candidate solutions are updated during iterations, the optimal solution obtained so far is also updated.

(3) $$F_{cranes}=\left[\begin{array}{c} F(X_{1})\\ \vdots \\ F(X_{i})\\ \vdots \\ F(X_{n}) \end{array}\right]=\left[\begin{array}{c} F(x_{1,1},\ldots ,x_{1,j},\ldots ,x_{1,d})\\ \vdots \\ F(x_{i,1},\ldots ,x_{i,j},\ldots ,x_{i,d})\\ \vdots \\ F(x_{n,1},\ldots ,x_{n,j},\ldots ,x_{n,d}) \end{array}\right],$$

### 2.3. Mathematical Model of RCO

In the RCO algorithm, the position update formulas of red-crowned cranes are established based on their behaviors of dispersing for foraging, avoiding danger, gathering for roosting, and dancing. Specifically, the following four rules are formulated:

1. Dispersing for foraging: The first thing to be pointed out is that the best position discovered by red-crowned cranes in the current iteration is considered an ideal habitat. Then, when daylight comes, the red-crowned cranes disperse from this habitat in search of food. They can be divided into two categories. Some red-crowned cranes forage randomly around the habitat, which are known as random foragers. Others have the courage to fly away from the habitat to explore richer food. These red-crowned cranes are called long-distance foragers.
2. Avoiding danger: For the long-distance foragers, they usually live on the edge of the population and are more likely to be exposed to danger. Therefore, these red-crowned cranes are very alert when foraging for food. As soon as danger is imminent, they emit a ‘ko-lo-lo-’ call and take to the air to escape from the danger.
3. Gathering for roosting: When the red-crowned cranes forage during the day, they also consider choosing a better habitat. If one red-crowned crane reaches a better position, this position will become a new habitat. At night, with the guidance of this red-crowned crane, other red-crowned cranes gather towards the new habitat.
4. Crane dance: With certain probability, a male red-crowned crane and a female red-crowned crane can successfully pair up and express their love for each other through singing, jumping, and dancing. During this time, they sing and make loud sounds. As a result, other red-crowned cranes stop near them to enjoy their dance. In this case, the two red-crowned cranes with the first and second fitness values are considered this pair of red-crowned cranes.

#### 2.3.1. Strategies Based on Foraging and Roosting Behaviors

First, according to the fitness values, the red-crowned cranes are divided into long-distance foragers and random foragers in a certain proportion. Specifically, the fitness values of the red-crowned cranes are sorted. The top-ranked red-crowned cranes are classified as random foragers, and the bottom-ranked red-crowned cranes are classified as long-distance foragers. Here, since red-crowned cranes with poor fitness values are often in danger, they are always alert, which gives them the courage to fly far away for food. The positions of the random foragers *X_rf_* and the long-distance foragers *X_lf_* are as follows:

(4) $$X_{rf}=\left[\begin{array}{c} X_{F1}\\ \vdots \\ X_{Fk} \end{array}\right]=\left[\begin{array}{ccccc} x_{F1,1} & \ldots & x_{F1,j} & \ldots & x_{F1,d}\\ \vdots & \vdots & \vdots & \vdots & \vdots \\ x_{Fk,1} & \ldots & x_{Fk,j} & \ldots & x_{Fk,d} \end{array}\right],$$

(5) $$X_{lf}=\left[\begin{array}{c} X_{F(k+1)}\\ \vdots \\ X_{Fn} \end{array}\right]=\left[\begin{array}{ccccc} x_{F(k+1),1} & \ldots & x_{F(k+1),j} & \ldots & x_{F(k+1),d}\\ \vdots & \vdots & \vdots & \vdots & \vdots \\ x_{Fn,1} & \ldots & x_{Fn,j} & \ldots & x_{Fn,d} \end{array}\right],$$

where *X_F_*_1_, …, *X_Fk_* are the positions of the first *k* red-crowned cranes after sorting the fitness values of all red-crowned cranes, and *X_F_*_(*k*+1)_, …, *X_Fn_* are the positions of the last (*n* − *k*) red-crowned cranes after sorting the fitness values of all red-crowned cranes. The new positions reached by random and long-distance foragers dispersing from the habitat to forage are shown in Equations (6) and (7), respectively.

(6) $$X_{i}^{0}(t+1)=X_{i}(t)+c_{1}\cdot R\cdot \left(X_{home}(t)-X_{i}(t)\right),i=F1,\ldots ,Fk,$$

(7) $$X_{i}^{0}(t+1)=X_{i}(t)+c_{2}\cdot \left(X_{home}(t)-X_{i}(t)\right),i=F(k+1),\ldots ,Fn,$$

where *X_i_*(*t*) is the position reached by the *i*-th red-crowned crane after *t* iterations, and *X_home_*(*t*) is the position of the ideal habitat obtained after *t* iterations, that is, the best position reached by the entire population of red-crowned cranes after *t* iterations. *R* is a 1 × *d* matrix, and its each element is a random number in the range of 0 to 1. *c*_1_ and *c*_2_ are the step adjustment coefficients. The step size *c*_1_ and parameter *R* determine the movement of random foragers. Since random foragers only search for food near the habitat, *c*_1_ is set to a constant value. However, if a large constant value of *c*_2_ is also used to force long-distance foragers to move, they will never be able to approach the optimal solution in the later stages of iteration. Therefore, we consider that *c*_2_ decreases linearly during the iteration process. Through the trial-and-error method, *c*_1_ is set to 2, and *c*_2_ is set to 5 − 4·*t*/*t*_max_, where *t*_max_ is the maximum number of iterations.

**Figure 2:**
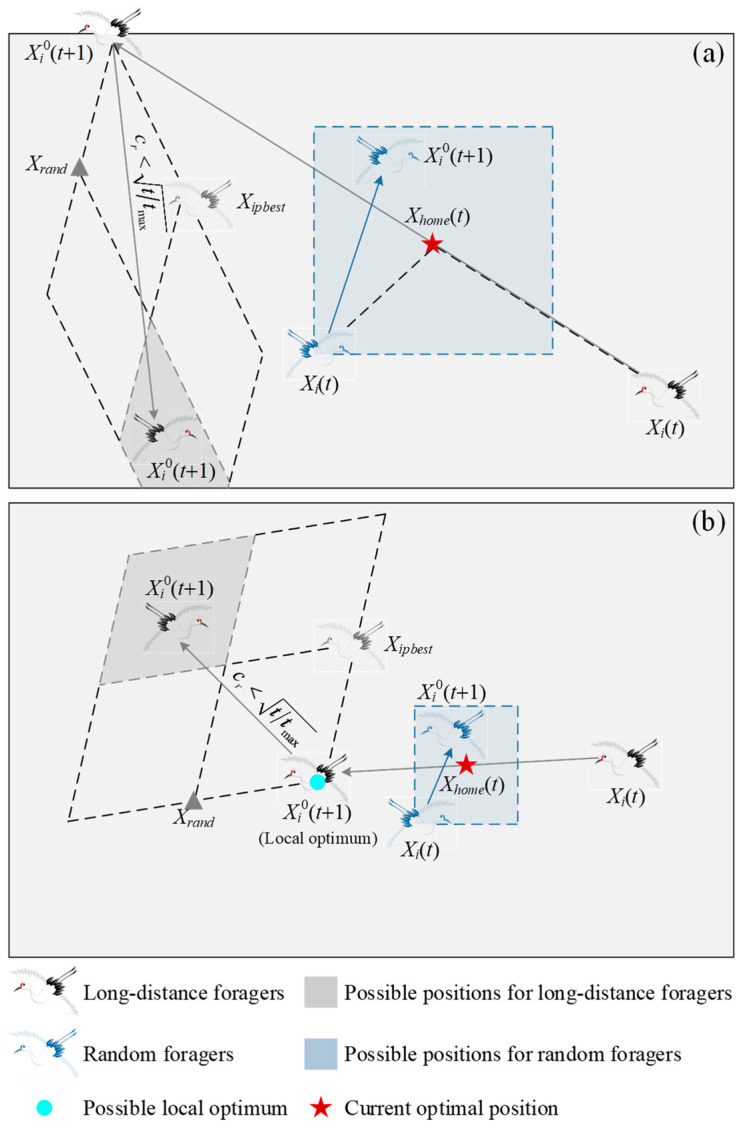
Schematic diagram of foraging and escaping behaviors of red-crowned cranes: (**a**) possible results in early iterations; (**b**) possible results in later iterations.

Figure 2 shows the schematic diagram (2D) of the foraging behavior of the red-crowned cranes, in which (a) and (b) are the possible results in the early and later iterations, respectively. It can be seen from Figure 2 that each random forager searches randomly within a rectangular range centered on the habitat, and the size of this range is determined by its distance from the habitat. Long-distance foragers fly along the habitat towards farther foraging grounds in the early iterations, but they still move within a certain area (search space). In the later phases, they move in smaller steps, either foraging or resting. As a result, in the early iteration phases, the red-crowned crane population has rich diversity, with both search agents surrounding current optimal position and search agents performing global searches. Obviously, the cooperation between random and long-distance foragers endows the algorithm with a certain local search capability during the exploration process, thereby accelerating convergence. As the number of iterations increases, the long-distance foragers gradually converge to current optimal positions. In addition, danger avoidance is an important strategy for long-distance foragers. Whether they are foraging or resting, they always keep looking up. When they realize that danger is coming, they quickly fly away from the dangerous position. It is assumed here that the probability of the appearance of danger increases with the increase in iterations. In other words, they have a high probability of changing the foraging grounds in the later iterations. This strategy can effectively prevent the algorithm from falling into local optimum, as shown in Figure 2b. Specifically, each long-distance forager has a risk coefficient *c_r_*, which is defined as a random value in the range of 0 to 1. When *c_r_* < (*t*/*t*_max_)^1/2^, the long-distance forager flies to a new position to replace the current foraging area, as follows:

(8) $$X_{i}^{0}(t+1)=X_{i}^{0}(t+1)+r_{1}\cdot \left(X_{rand}-X_{i}^{0}(t+1)\right)+r_{2}\cdot \left(X_{ipbest}-X_{i}^{0}(t+1)\right),\;\;\;i=F(k+1),\ldots ,Fn,$$

where *X_rand_* is a newly generated random position in the search space, *X_ipbest_* is the individual optimal position discovered by long-distance foragers in the past foraging process, which is different from the individual optimal position used in the PSO algorithm [11], and *r*_1_ and *r*_2_ are random numbers belonging to the range of 1 to 2.

When night falls, the red-crowned cranes fly back to their habitat one after another. Note that their habitat is not fixed every day. While foraging during the day, they also try to look for a better habitat. Therefore, the red-crowned crane that finds a better position after foraging guide other companions (including all random and long-distance foragers) to gather towards it. If no better position is found, all red-crowned cranes will still return to their original habitat. The behavior of gathering for roosting can be modeled as follows:

(9) $$X_{i}(t+1)=X_{i}^{0}(t+1)+c_{3}\cdot r_{3}\cdot \left(X_{home}^{0}(t+1)-X_{i}^{0}(t+1)\right),$$

where *X*^0^*_home_*(*t* + 1) is the habitat tonight, which may be updated or unchanged. *c*_3_ is a time-varying step adjustment coefficient, equal to 2 − *t*/*t*_max_, and *r*_3_ is a random value in the range of 0 to 1. The design of *c*_3_ is also to ensure a balance between exploration and exploitation.

**Figure 3:**
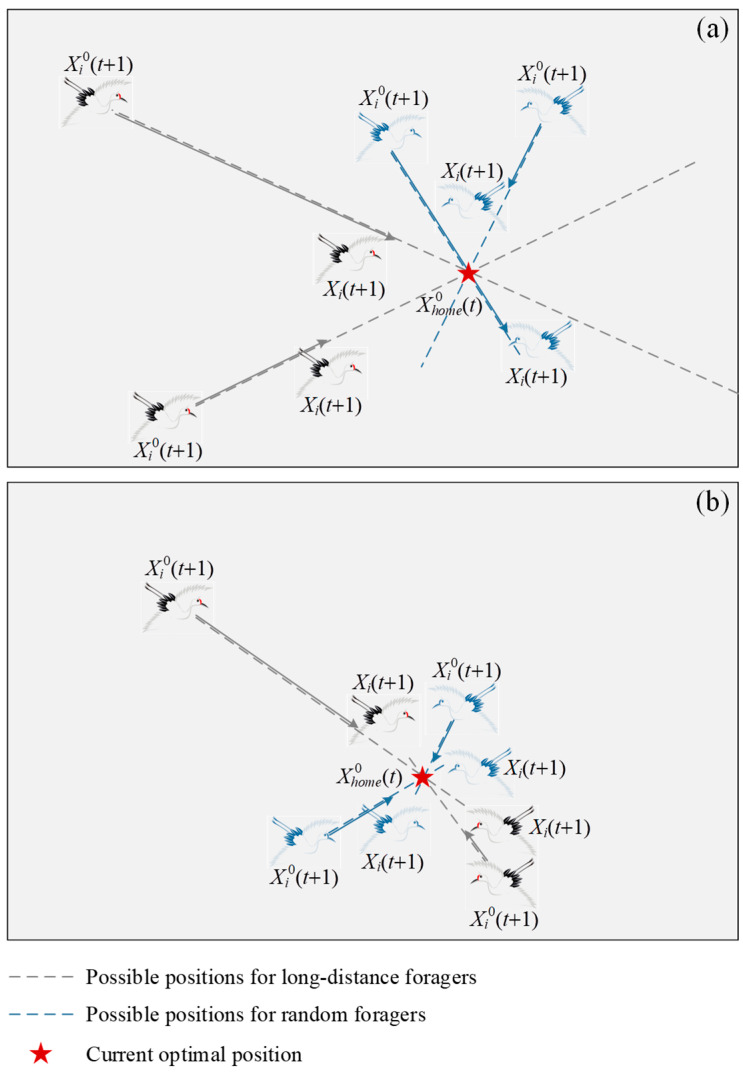
Schematic diagram of gathering behavior of red-crowned cranes: (**a**) possible results in early iterations; (**b**) possible results in later iterations.

Figure 3 shows the schematic diagram (2D) of the gathering behavior of red-crowned cranes. From Figure 3, a new habitat *X*^0^*_home_*(*t* + 1) is discovered, and then other red-crowned cranes gradually move there. They fly to the habitat in a straight direction and then randomly select a position to roost, which may be close to the habitat or far away. This is due to the fact that red-crowned cranes not only roost in habitats but may also choose to roost in foraging areas where food is abundant. The step coefficient *c*_3_ adopts a design that linearly decreases from 2 to 1 with the increase in iterations, which can reduce the probability of the red-crowned cranes moving away from the habitat and thus enables them to gradually converge to the habitat.

#### 2.3.2. Strategy Based on Crane Dance

This section establishes an update strategy based on the dancing behavior of the red-crowned cranes. In this case, a male crane and a female crane successfully pair up and engage in a series of interactions. As a result, other red-crowned cranes gather near them to watch their dance. Assume that the search agents with the first fitness value and the second fitness value are considered the pair of red-crowned cranes. Then, the position update formula of the red-crowned cranes can be modeled as follows:

(10) $${X_{i}}^{\prime }(t+1)=X_{i}(t)+u\cdot \left(r_{4}\cdot X_{first}(t)-X_{i}(t)\right),$$

(11) $${X_{i}}^{\prime \prime }(t+1)=X_{i}(t)+u\cdot \left(r_{4}\cdot X_{second}(t)-X_{i}(t)\right),$$

(12) $$X_{i}(t+1)=\frac{{X_{i}}^{\prime }(t+1)+{X_{i}}^{\prime \prime }(t+1)}{2},$$

where *r*_4_ is the deviation coefficient, and it is a random number in the range of 0 to 0.1. *X_first_*(*t*) and *X_second_*(*t*) correspond to the global optimum position and global second position after *t* iterations, respectively. *u* is a random number that obeys Gaussian distribution, as follows:

(13) $$u\sim N(1,\sigma_{u}^{2}),\sigma_{u}=1-\frac{t}{t_{\max}},$$

**Figure 4:**
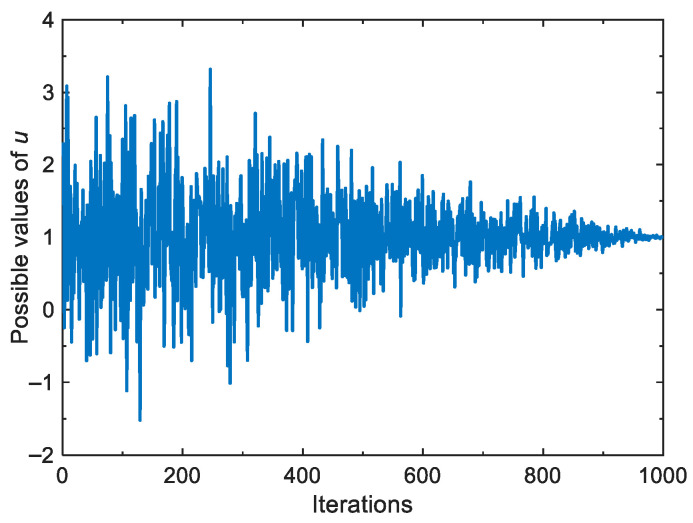
Possible values of the random number *u* during iterations.

Under this strategy, other red-crowned cranes update their positions based on the positions of the pair of red-crowned cranes that are dancing. The design of the deviation coefficient *r*_4_ is intended to prevent the algorithm from being over-exploited during iterations. From Equation (13), it is found that the dispersion degree of the Gaussian distribution is adaptively adjusted due to the gradual decrease in *σ_u_* from 1 to 0. This can promote the algorithm to have a certain global search capability in the early iterations, and in the later iterations, *u* is almost close to 1, thus giving the algorithm a good exploitation capability. Figure 4 shows the variation in the random number *u* during iterations.

### 2.4. Implementation of RCO

In each iteration of the RCO algorithm, the position update strategy developed based on foraging and roosting behaviors or the position update strategy developed based on crane dance is selected with a certain probability. Specifically, a probability coefficient *p_c_* is defined, which is a constant value in the range of 0.1 to 0.9. When a random number *r*_5_ in the range of 0 to 1 is less than *p_c_*, the position update strategy in Section 2.3.1 is selected in this iteration; otherwise, the position update strategy in Section 2.3.2 is selected. Regarding the parameter *p_c_*, we analyze and clarify it in the subsequent section, Section 3.2.5. Therefore, the internal parameters that need to be set for the RCO algorithm include the probability coefficient *p_c_* and the ratio of the random foragers to the long-distance foragers. The run of the algorithm is stopped based on the number of iterations *t* or the number of function evaluations *Fes*; that is, the external parameter of maximum number of iterations *t*_max_ or the maximum number of function evaluations *FE*_max_ also needs to be given. In addition, the size *n* of the red-crowned crane population also belongs to an external parameter. Algorithm 1 shows the pseudo-code of the proposed RCO algorithm.
**Algorithm 1:** Pseudo-code of RCO**Input:** The maximum number of iterations *t*_max_, the maximum number of function evaluations *FE*_max_, the population size *n*, the probability coefficient *p_c_*, and the ratio of random foragers to long-distance foragers *k*:(*n*-*k*); **Output:** The best solution *X_best_* and its fitness value *F*(*X_best_*). 1:   Initialize the red-crowned cranes *X_cranes_* using Equations (1) and (2) 2:   *t* = 0 and *FEs* = 0 3:   **while** (*t* < *t*_max_ or *FEs* < *FE*_max_) 4:    Calculate the fitness values of all red-crowned cranes using Equation (3) 5:    Record the first and second individuals so far 6:    **if** *r*_5_ < *p_c_* 7:     Take the position corresponding to the first fitness value as *X_home_* 8:     Sort the red-crowned cranes according to their fitness values 9:     **for** *i* = *F*1:*Fk  /Foraging behavior of random foragers/* 10:      Update the positions of the random foragers using Equation (6) 11:      Calculate the fitness values of random foragers 12:      **end for** 13:      **for** *i* = *F*(*k*+1):*Fn  /Foraging behavior of long-distance foragers/* 14:       Update the positions of the long-distance foragers using Equation (7) 15:       **if** *c_r_* < (*t*/*t*_max_)^1/2^  */Escaping behavior of long-distance foragers/* 16:        Generate *X_rand_* and record *X_ipbest_* of long-distance foragers 17:        Further update their positions using Equation (8) 18:       **end if** 19:       Calculate the fitness values of long-distance foragers 20:      **end for** 21:      Determine *X_home_* by comparing the fitness values of all red-crowned cranes after foraging with the fitness values of *X_home_* 22:      **for** *i* = 1:*n  /Roosting behavior of red-crowned cranes/* 23:       Update the positions of all red-crowned cranes using Equation (9) 24:      **end for** 25:      *FEs* = *FEs* + 2*n* 26:     **else** 27:      **for** *i* = 1:*n  /Crane dance of red-crowned cranes/* 28:       Update the positions of red-crowned cranes using Equation (12) 29:      **end for** 30:      *FEs* = *FEs* + *n* 31:     **end if** 32:     *t* = *t* + 1 33: **end while** 34: Return *X_best_* and *F*(*X_best_*)

### 2.5. Computational Complexity of RCO

The computational complexity of RCO is mainly affected by the population initialization, the fitness evaluation, and the position update. During the initialization process, the computational complexity is *O*(*n*) due to the use of *n* search agents. When the foraging and roosting strategies are used to update the positions, a quick sort on the fitness values is required, and its computational complexity is *O*(*n*log_2_*n*). In addition, all search agents perform the foraging and roosting phases successively, with a computational complexity of *O*(2*nd* + 2*n*). Then, the computational complexity of executing this strategy once is *O*(*n*log_2_*n* + 2*nd* + 2*n*). The computational complexity of using the crane dance strategy is *O*(*nd* + *n*). In the iteration, the probability of using the former strategy is *p_c_*, and the probability of using the latter strategy is (1 − *p_c_*). Finally, the total computational complexity is

(14) $$\begin{array}{l} \;\;\;\;O\left(n+p_{c}t_{\max}(n\log_{2}n+2nd+2n)+(1-p_{c})t_{\max}(nd+n)\right)\\ =O\left(n+p_{c}t_{\max}n\log_{2}n+(1+p_{c})t_{\max}n(d+1)\right) \end{array},$$

## 3. Experimental Results and Discussion

### 3.1. Experimental Setup

To demonstrate the performance of the proposed algorithm, this section tests RCO and eight other algorithms on two types of test function sets (CEC-2005 functions [49] and CEC-2022 functions [50]). The eight algorithms for comparison are as follows: Rüppell’s fox optimizer (RFO) [51], Eel and Grouper Optimizer (EGO) [52], Coati Optimization Algorithm (COA) [53], Harris Hawks Optimizer (HHO) [33], Slime Mould Algorithm (SMA) [42], Runge–Kutta Optimizer (RUN) [54], Golden Jackal Optimization (GJO) [41], and Dung Beetle Optimizer (DBO) [34]. Table 2 presents the internal parameter settings of the RCO algorithm and other comparative algorithms, with parameters set according to their original texts. For the CEC-2005 functions, all algorithms are run with 50,000 function evaluations and 50 search agents. Similarly, for the CEC-2022 functions, *FEs* and *n* are set to 100,000 and 50, respectively. To ensure more reliable results, all algorithms are run 30 times. Experiments for all algorithms are conducted using MATLAB 2021a on an Intel Core i7-10750H Processor with 2.60 GHz and 16.0 GB of main memory.

### 3.2. Tests on CEC-2005 Benchmark Functions

#### 3.2.1. Exploitation and Exploration Analysis

The CEC-2005 test functions contain seven variable-dimensional unimodal functions (*F*_1_–*F*_7_), six variable-dimensional multimodal functions (*F*_8_–*F*_13_), and ten fixed-dimensional multimodal functions (*F*_14_–*F*_23_) [49]. Here, the dimensions of *F*_1_–*F*_13_ are fixed to 30. The unimodal functions can be used to test the exploitation capacity of the algorithm since they have only one global optimal solution, while the multimodal functions with local optimal solutions are used to examine the exploration capacity of the algorithm. Compared with multimodal functions *F*_8_–*F*_13_, functions *F*_14_–*F*_23_ are easier to solve due to their lower dimensionality and fewer local optimal solutions. The performance of all optimizers on these functions is presented through mean, standard deviation, minimum, and maximum values of the results of 30 independent runs, and the ranking (algorithms with the same performance share the sum of their rankings equally), as shown in Table A1 of Appendix A.

For the unimodal functions *F*_1_ and *F*_3_, the RCO, SMA, and EGO algorithms successfully obtain the optimal value (i.e., zero) in every run, which is significantly better than the other algorithms. For functions *F*_2_, *F*_4_, and *F*_7_, the RCO algorithm also provides better mean and standard deviation results than all other algorithms. For *F*_6_, RCO ranks third in terms of mean and standard deviation, after DBO and RUN. Overall, the RCO algorithm ranks first in functions *F*_1_, *F*_2_, *F*_3_, *F*_4_, and *F*_7_, and the test results of these unimodal functions demonstrate the superior exploitation capability of RCO.

The optimization process for the multimodal functions *F*_9_, *F*_10_, and *F*_11_ seems to be easy, and RCO, DBO, RUN, SMA, HHO, COA, and EGO can make them converge to the optimum in all runs. For function *F*_12_, the RCO optimizer gives satisfactory results and is slightly inferior to the RUN optimizer in terms of the mean and standard deviation values, ranking second. Observing the performance of RCO in functions *F*_14_–*F*_23_, it can be found that although some competing optimizers also provide the same mean values as RCO, they give clearly worse standard deviation results. In addition, it can be judged from the index of the maximum value that DBO, GJO, HHO, COA, and EGO have relatively weak exploration capabilities and are prone to falling into local optima. In contrast, RUN and SMA have better exploration performance. There is no doubt that the RCO optimizer shows significant exploration capability and strong competitiveness for multimodal functions.

From the ranking point of view, the RCO algorithm ranks first (or tied for first) in five of the seven unimodal functions, and ranks first (or tied for the first) in twelve of the sixteen multimodal functions. Therefore, the proposed RCO algorithm exhibits better optimization accuracy on 74% of the CEC-2005 benchmark functions. This indicates that the RCO algorithm outperforms the other compared algorithms in optimizing the benchmark functions and ranks first overall.

#### 3.2.2. Convergence Analysis

**Figure 5:**
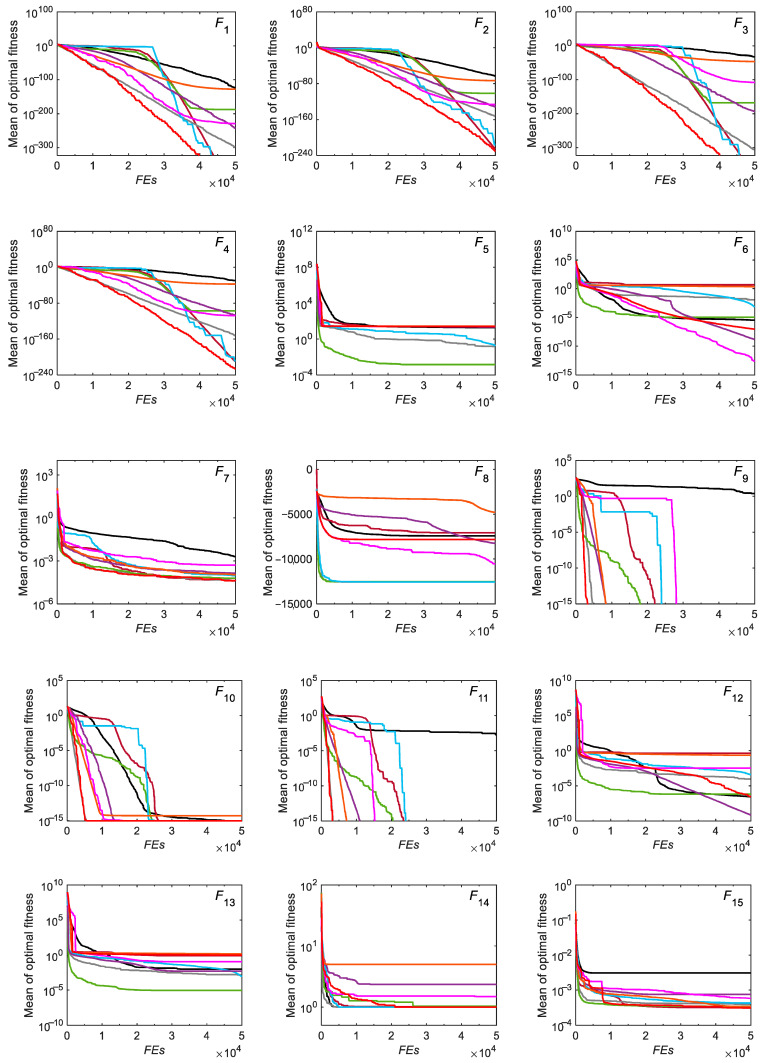

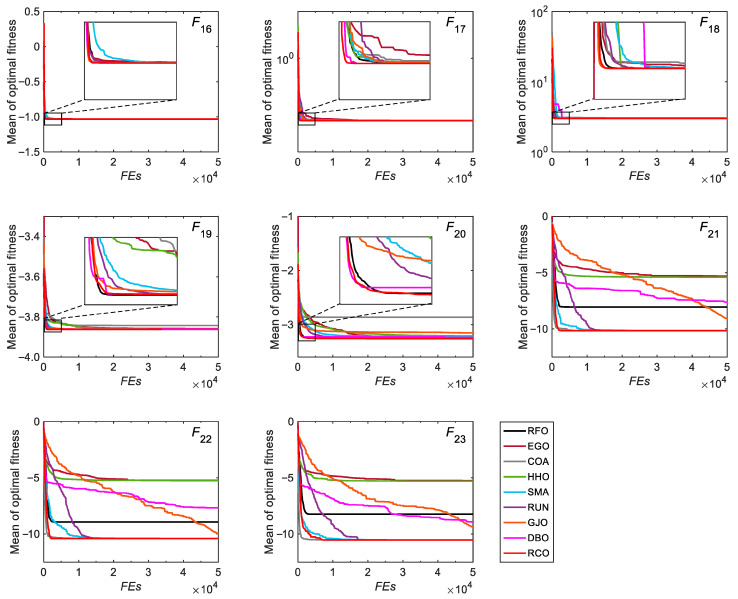
Convergence curves for CEC-2005 functions optimized by RCO and other optimizers.

In order to intuitively analyze the convergence speed of the RCO algorithm, the convergence curves of the RCO algorithm and other algorithms on the benchmark test functions are drawn, as shown in Figure 5. Under the same population size and number of function evaluations, RCO is able to achieve better results more efficiently than other optimizers for the unimodal functions *F*_1_, *F*_2_, *F*_3_, *F*_4_, and *F*_7_, showing fast convergence speed and high convergence accuracy. For the multimodal test functions *F*_9_–*F*_11_ and *F*_16_–*F*_23_, RCO can effectively avoid falling into the local optimum by virtue of its promising exploration capability, thereby allowing these functions to quickly converge to the global optimum. Although some competing algorithms can also make these functions converge to similar values, their convergence speed obviously fails to exceed that of the RCO algorithm, which is relatively slow. In general, the convergence speed of the RCO algorithm is satisfactory.

#### 3.2.3. Non-Parametric Statistical Analysis

To further evaluate the performance of the RCO algorithm, the Wilcoxon signed-rank test at *α* = 0.05 level is used to determine whether there is a significant difference in performance of RCO compared to its competitors. Table 3 gives the statistical results of RCO compared to its competitors one by one. Note that “+” means that there is a significant difference between the RCO algorithm and competitors at the 0.05 level, and RCO outperforms its competitors. “−” likewise means that there is a significant difference between RCO and competitors, but RCO is not as good as its competitors. “=” indicates that there is no significant difference between the two.

RCO significantly outperforms GJO on 20 functions, outperforms HHO, COA, and EGO on 16 functions, and outperforms RUN on 15 functions. Furthermore, it outperforms DBO and SMA on 13 functions. However, it is significantly inferior to other algorithms on at most four functions. This indicates that compared with other algorithms, the RCO algorithm achieves significant improvements in optimization performance. From the statistical results on unimodal functions alone, RCO outperforms GJO on all functions, outperforms DBO and RFO on six functions, and outperforms RUN, HHO, and COA on five functions. However, the comparison between RCO and SMA shows four “=”, indicating that there is no significant difference between them. In summary, the RCO algorithm exhibits significantly better exploitation ability compared with other algorithms except SMA. Finally, on multimodal functions, the RCO algorithm obtains at least 10 “+” and at most 3 “−” compared with all other algorithms except DBO and RFO, which means that it also has significant advantages in exploration ability. It is worth mentioning that there is no significant difference between RCO and RFO on most (10) multimodal functions. Overall, it can be concluded that the proposed algorithm has a significantly superior comprehensive optimization ability.

#### 3.2.4. Scalability Analysis

**Figure 6:**
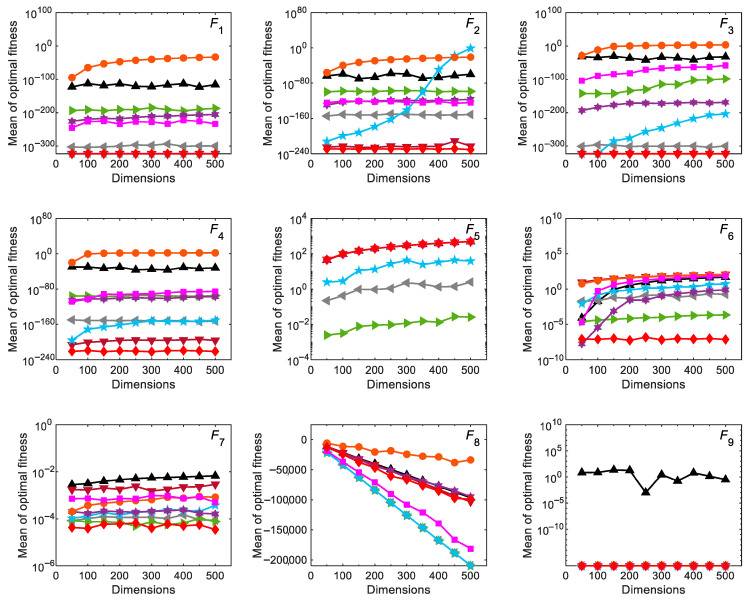

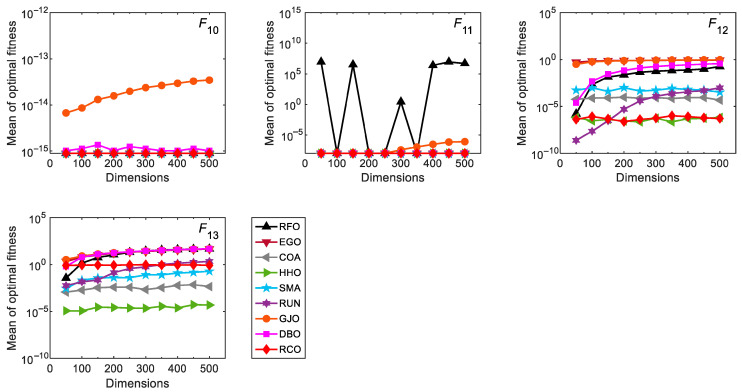
Scalability results for scalable functions (*F*_1_–*F*_13_) optimized by RCO and other optimizers in the case of different dimensions.

Scalability analysis is used to show whether the algorithms perform similarly for low-dimensional and high-dimensional tasks. In this test, the dimensions of the variable-dimensional functions *F*_1_–*F*_13_ vary from 50 to 500 with a step size of 50. Figure 6 shows the results of the scalability test for all optimization algorithms. It can be observed that the performance of RCO, HHO, and COA does not decrease significantly as the variable dimension increases, and they basically show consistent search capabilities for all functions. The optimization capabilities of DBO, GJO, RUN, SMA, EGO, and RFO for some functions decrease with the increase in dimensions. It should be emphasized that the function *F*_8_ exhibits a downward trend since its optimal value decreases linearly as the dimension increases. To sum up, the variable dimensions have less impact on the solution quality of the RCO HHO, and COA than the other optimization algorithms. Table 4 gives the evaluation results of all algorithms optimizing functions *F*_1_–*F*_13_ in 500 dimensions. It is found that RCO provides the best results for functions *F*_1_–*F*_4_, *F*_6_–*F*_7_, and *F*_9_–*F*_12_ (the ones tied for first place are also taken into account), and the differences between these results and the results of *F*_1_–*F*_13_ in 30 dimensions are small. It reveals that RCO has good scalability due to its ability to provide satisfactory results for high-dimensional problems.

#### 3.2.5. Parameter Analysis

**Figure 7:**
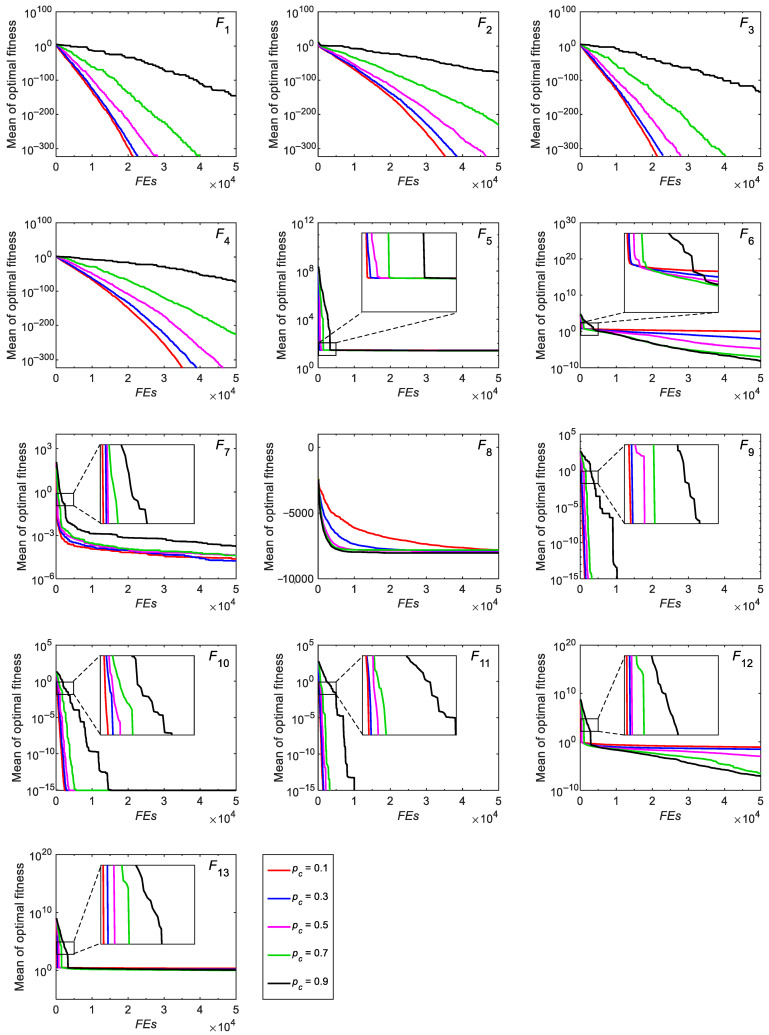
Convergence curves for benchmark test functions optimized by RCO in the case of different probability coefficients.

This section focuses on exploring the effect of the parameter *p_c_* on the performance of the RCO algorithm. Table 5 shows the mean and standard deviation values of 30 results when the probability coefficient *p_c_* of RCO is 0.1, 0.3, 0.5, 0.7, and 0.9. From Table 5, it is found that for unimodal functions except *F*_6_, the smaller the parameter *p_c_* is, the better the results are, which is particularly obvious for functions *F*_1_–*F*_4_. For multimodal functions *F*_8_, *F*_12_, and *F*_13_, as the probability coefficient pc increases, RCO provides better results. Moreover, for multimodal functions *F*_14_–*F*_23_, it is observed that the standard deviation value decreases with the increase in the probability coefficient *p_c_*, which means that the obtained results are more stable. There is no doubt that the probability coefficient significantly affects the exploitation and exploration performance of the algorithm. Specifically, a smaller pc value can enhance the exploitation capability of the RCO algorithm, and a larger *p_c_* value endows RCO with a superior exploration capability. Figure 7 shows the convergence curves of RCO for functions *F*_1_–*F*_13_ under different *p_c_* values. It can be found that, except for function *F*_8_, RCO has the fastest convergence speed when *p_c_* is 0.1 and the slowest convergence speed when *p_c_* is 0.9. Therefore, the RCO convergence speed can be improved by reducing the value of *p_c_*.

#### 3.2.6. Running Time Comparison

Table A2 in Appendix A presents the average running time of each algorithm on the CEC-2005 benchmark functions. Among all the algorithms, EGO has the shortest computational time, while RUN and SMA have relatively longer computational times. Moreover, all other algorithms, including RCO, have similar running time. Therefore, the time metrics indicate that the proposed RCO algorithm improves optimization capability without significantly increasing the computational time.

### 3.3. Tests on CEC-2022 Functions

This section uses the newer CEC-2022 test function set to further verify the search performance of RCO. It contains a total of twelve functions in four categories: unimodal, multimodal, hybrid, and composite. During the test, the dimensions of all functions are set to 10. Table A3 of Appendix A gives the mean, standard deviation, minimum, maximum, and ranking results of all algorithms for these functions. For function *F*_24_, RCO, RUN, and SMA provide superior results. Further comparison shows that the standard deviation value of RCO is smaller than that of RUN and SMA. For the multimodal functions *F*_25_–*F*_28_, RCO ranks first for two functions and ranks third after SMA and RFO for the other two. For two of the three hybrid functions *F*_29_–*F*_31_, the performance of RCO is poor and only at an intermediate level. In addition, RCO is also highly competitive in terms of composition functions *F*_32_–*F*_35_. These results show that the RCO algorithm is feasible and exhibits overall better performance than the compared algorithms on the CEC-2022 test set.

In addition, the Wilcoxon signed-rank test is also used for further comparison, and the statistical results are shown in Table 6. Obviously, RCO is superior to GJO, COA, EGO, and RFO in most functions. Compared with DBO and HHO, RCO outperforms them in half of the functions, and shows no significant differences in other functions. There are significant differences between RCO and RUN for six functions, but RCO is surpassed by RUN in two functions. RCO is better than SMA for five functions and worse than SMA for five functions. Overall, from a statistical significance perspective, RCO is rarely surpassed by other algorithms, as “−” appears only 9 times out of 96 (8 × 12) comparisons. In addition, “+” appears 54 times, and “=” appears 33 times. This further demonstrates that RCO can effectively achieve the optimization of the CEC-2022 test set and indicate its certain advantages.

## 4. Application of RCO in Engineering Design Problems

To verify the ability of the proposed RCO algorithm to solve actual engineering problems, RCO and fifteen other optimizers (Rüppell’s fox optimizer (RFO) [51], the Eel and Grouper Optimizer (EGO) [52], Coati Optimization Algorithm (COA) [53], Artificial Bee Colony (ABC) [12], Moth–Flame Optimization (MFO) [32], Ant Lion Optimizer (ALO) [55], Multi-Verse Optimizer (MVO) [24], Dragonfly Algorithm (DA) [17], Grasshopper Optimisation Algorithm (GOA) [56], Seagull Optimization Algorithm (SOA) [57], Harris Hawks Optimizer (HHO) [33], Slime Mould Algorithm (SMA) [42], Runge–Kutta Optimizer (RUN) [54], Golden Jackal Optimization (GJO) [41], and Dung Beetle Optimizer (DBO) [34]) are used to optimize eight reported classic engineering design problems. These optimization problems usually have some constraints, and the values of their decision variables are not always continuous, which are challenges for the optimizer. Note that each optimizer is run 30 times in optimizing each problem and uses 50 individuals (*n*) and 50,000 function evaluations (*FEs*). The parameters for RCO are as follows: *p_c_* = 0.9, *k*:(*n* − *k*) = 1:1. The parameters for the other optimizers follow the directions set out the original paper. The performance of RCO and other optimizers is evaluated based on four statistical indicators (mean, standard deviation, and best and worst values) and convergence curves. These problems include a three-bar truss design [58], cantilever beam problem [59], corrugated bulkhead problem [60], speed reducer problem [61]), Himmelblau’s nonlinear problem [62], I-beam problem [63], tension/compression spring problem [64], and reinforced concrete beam problem [65]. For the sake of conciseness in the main text, the results and analyses of the latter four problems are provided in Appendix B.1, Appendix B.2, Appendix B.3 and Appendix B.4.

### 4.1. Constraint Handling Method

In optimization problems with constraints, penalty function methods are an effective means to handle constraints. There are many penalty methods, and some common methods have been summarized in the literature [66]. The death penalty and static penalty are the two most popular methods. This paper chooses the latter to handle constraints in engineering design problems, which is achieved by constructing the following static penalty function:

(15) $$F^{\prime }(X)=F(X)+\sum_{i=1}^{m}\left(l_{i}\cdot \max{\left[0,g_{i}(X)\right]}^{2}\right),$$

where *F′*(*X*) is the modified objective function, *l_i_* (*I* = 1, 2, …, *m*, and *m* is the number of constraints for the problem to be solved) are the penalty factors, and *g_i_*(*X*) are the values of the constraint functions. In this paper, *l_i_* are assigned large values.

### 4.2. Three-Bar Truss Design Problem

**Figure 8:**
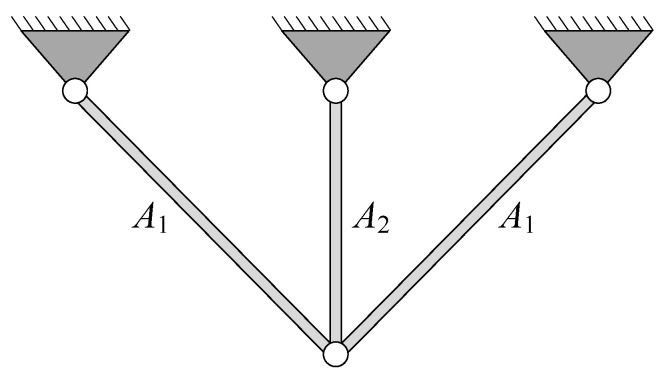
Structure diagram of three-bar truss.

In the three-bar truss design problem, it is necessary to consider reducing the volume as much as possible while satisfying the stress constraints on each side of the member. The two variables of this problem are the cross-sectional areas *A*_1_ and *A*_2_ of the bars, as shown in Figure 8. Specifically, this problem can be expressed as follows:

Consider variable $X=\left[x_{1},x_{2}\right]=\left[A_{1},A_{2}\right]$;

minimize $F(X)=\left(2\sqrt{2}x_{1}+x_{2}\right)l$;

subject to

$$g_{1}(X)=\frac{\sqrt{2}x_{1}+x_{2}}{\sqrt{2}x_{1}^{2}+2x_{1}x_{2}}P-\sigma \leq 0,$$

$$g_{2}(X)=\frac{x_{2}}{\sqrt{2}x_{1}^{2}+2x_{1}x_{2}}P-\sigma \leq 0,$$

$$g_{3}(X)=\frac{1}{x_{1}+\sqrt{2}x_{2}}P-\sigma \leq 0;$$

where l=100 cm, $P=2\;\mathrm{kN}/{\mathrm{cm}}^{2}$, $\sigma =2\;\mathrm{kN}/{\mathrm{cm}}^{2}$;

variable range: $0\leq x_{1},x_{2}\leq 1$.

**Figure 9:**
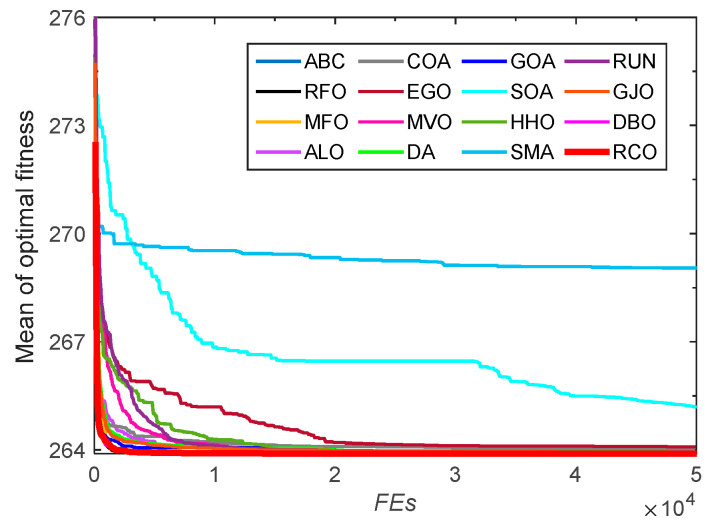
Convergence curves for three-bar truss design problem optimized by RCO and other optimizers.

Table 7 shows the results for the three-bar truss problem using sixteen optimizers. In terms of best value and mean value, DBO ranks first. It is worth mentioning that RCO finds the best value very close to that found by DBO and exceeds other algorithms in terms of mean value. The best solution obtained by RCO is *X* = (0.788633343920, 0.408366505177). Figure 9 shows the convergence curves for all methods. Obviously, RCO has a very satisfactory convergence performance in dealing with this problem.

### 4.3. Cantilever Beam Design Problem

**Figure 10:**
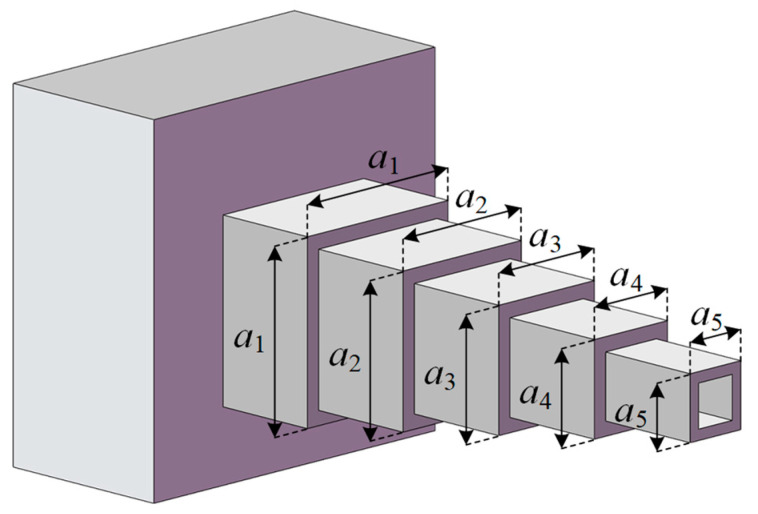
Structure diagram of cantilever beam.

Figure 10 shows a cantilever beam composed of five hollow square tubes with constant thickness. The width and height of each hollow tube are equal. The widths (heights) of the five hollow tubes are considered as the five variables in the design of the cantilever beam. In the cantilever beam problem, the goal is to minimize the weight of the entire cantilever beam. This problem can be expressed by the following formula:

Consider variable $X=\left[x_{1},x_{2},x_{3},x_{4},x_{5}\right]=\left[a_{1},a_{2},a_{3},a_{4},a_{5}\right]$;

minimize $F(X)=0.0624\left(x_{1}+x_{2}+x_{3}+x_{4}+x_{5}\right)$;

subject to

$$g_{1}(X)=\frac{61}{x_{1}^{3}}+\frac{37}{x_{2}^{3}}+\frac{19}{x_{3}^{3}}+\frac{7}{x_{4}^{3}}+\frac{1}{x_{5}^{3}}-1\leq 0;$$

variable range: $0.01\leq x_{1},x_{2},x_{3},x_{4},x_{5}\leq 100$.

**Figure 11:**
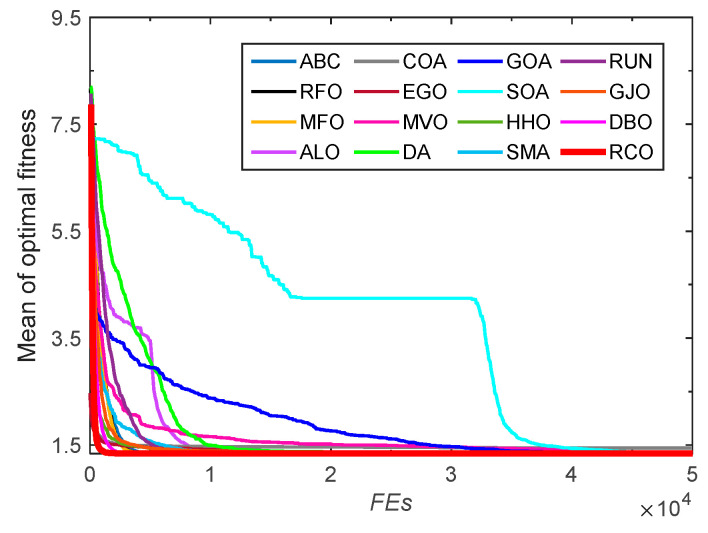
Convergence curves for cantilever beam design problem optimized by RCO and other optimizers.

Table 8 shows the detailed results of various indicators when optimizing the cantilever beam problem. It is observed that DBO gives the first ranked mean, standard deviation, and best values, followed by RUN. RCO ranks third, but the difference between the best value returned by RCO and that returned by DBO or RUN is very small. The solution corresponding to the minimum weight of the cantilever beam achieved by RCO is *X* = (6.016442523051, 5.308074580329, 4.491372442055, 3.500315808517, 2.157480922246). In addition, it is found from Figure 11 that RCO converges to a good result at the earliest.

### 4.4. Corrugated Bulkhead Design Problem

**Figure 12:**
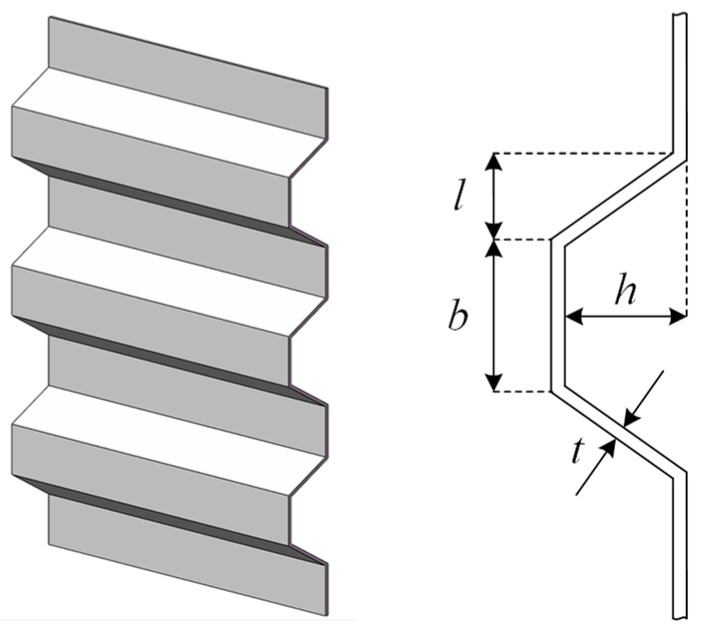
Structure diagram of corrugated bulkhead.

In the corrugated bulkhead design problem, the weight of the bulkhead is required to be minimized. As shown in Figure 12, the bulkhead has four variable parameters, which are width *b*, depth *h*, length *l*, and thickness *t*. Additionally, the problem is subject to six inequalities. The mathematical model of this minimization problem is as follows:

Consider variable $X=\left[x_{1},x_{2},x_{3},x_{4}\right]=\left[b,h,l,t\right]$;

minimize $F(X)=\frac{5.885\left(x_{1}+x_{3}\right)x_{4}}{x_{1}+\sqrt{\left|x_{3}^{2}-x_{2}^{2}\right|}}$;

subject to

$$g_{1}(X)=-x_{2}x_{4}\left(\frac{2x_{1}}{5}+\frac{x_{3}}{6}\right)+8.94\left(x_{1}+\sqrt{\left|x_{3}^{2}-x_{2}^{2}\right|}\right)\leq 0,$$

$$g_{2}(X)=-x_{2}^{2}x_{4}\left(\frac{x_{1}}{5}+\frac{x_{3}}{12}\right)+2.2{\left[8.94\left(x_{1}+\sqrt{\left|x_{3}^{2}-x_{2}^{2}\right|}\right)\right]}^{\frac{4}{3}}\leq 0,$$

$$g_{3}(X)=0.0156x_{1}-x_{4}+0.15\leq 0,$$

$$g_{4}(X)=0.0156x_{3}-x_{4}+0.15\leq 0,$$

$$g_{5}(X)=-x_{4}+1.05\leq 0,$$

$$g_{6}(X)=x_{2}-x_{3}\leq 0;$$

variable range: $0\leq x_{1},x_{2},x_{3}\leq 100$, $0\leq x_{4}\leq 5$.

**Figure 13:**
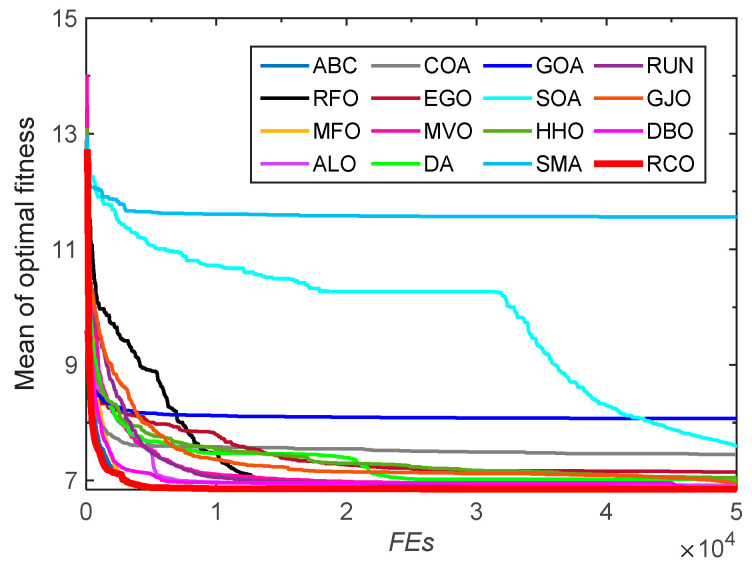
Convergence curves for corrugated bulkhead design problem optimized by RCO and other optimizers.

From the statistical results shown in Table 9, it is found that RCO, DBO, MFO, and RFO provide the same best value, and this value is the optimal objective function value. The optimal solution obtained is *X* = (57.692307672839, 34.147620293494, 57.692307345992, 1.050000000008). Among these three optimization algorithms, MFO ranks first due to its lower mean and standard deviation values, RFO ranks second only to MFO, and RCO ranks third. Finally, Figure 13 shows that RCO outperforms other algorithms in terms of convergence performance.

### 4.5. Speed Reducer Design Problem

**Figure 14:**
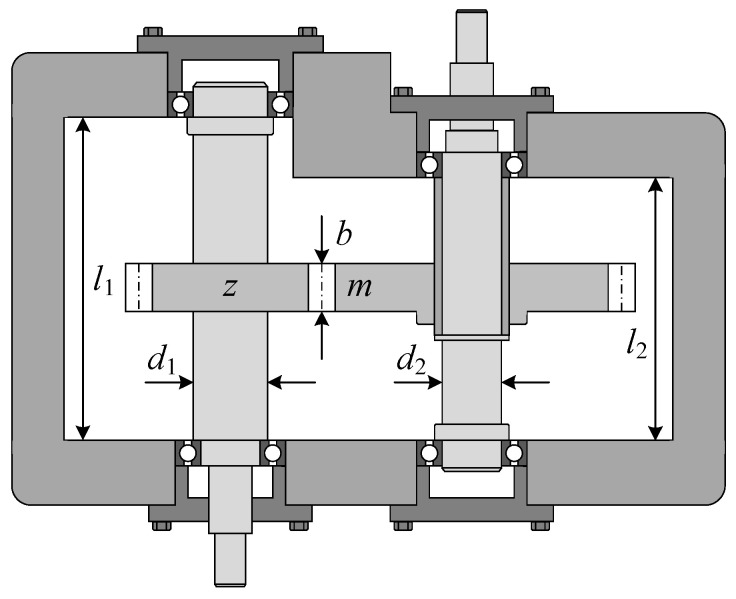
Structure diagram of speed reducer.

Speed reducers are important transmission components in the industrial field. In the speed reducer optimization problem, it is required to minimize the weight of the speed reducer while satisfying four linear and seven nonlinear constraints. The problem has seven variables: the face width *b*, the module of teeth *m*, the number of teeth in the pinion *z*, the length of the first shaft between bearings *l*_1_, the length of the second shaft between bearings *l*_2_, the diameter of the first shaft *d*_1_, and the diameter of the second shaft *d*_2_. Note that the variable *z* can only be set to an integer; that is, it is a discrete variable. Figure 14 shows the schematic diagram of a speed reducer. The problem is clearly expressed as follows:

Consider variable $X=\left[x_{1},x_{2},x_{3},x_{4},x_{5},x_{6},x_{7}\right]=\left[b,m,z,l_{1},l_{2},d_{1},d_{2}\right]$;

minimize $\begin{array}{ll} F(X)= & 0.7854x_{1}x_{2}^{2}\left(3.3333x_{3}^{2}+14.9334x_{3}-43.0934\right)\\ & -1.508x_{1}\left(x_{6}^{2}+x_{7}^{2}\right)+7.4777\left(x_{6}^{3}+x_{7}^{3}\right)+0.7854\left(x_{4}x_{6}^{2}+x_{5}x_{7}^{2}\right) \end{array}$;

subject to

$$g_{1}(X)=\frac{27}{x_{1}x_{2}^{2}x_{3}}-1\leq 0,$$

$$g_{2}(X)=\frac{397.5}{x_{1}x_{2}^{2}x_{3}^{2}}-1\leq 0,$$

$$g_{3}(X)=\frac{1.93x_{4}^{3}}{x_{2}x_{3}x_{6}^{4}}-1\leq 0,$$

$$g_{4}(X)=\frac{1.93x_{5}^{3}}{x_{2}x_{3}x_{7}^{4}}-1\leq 0,$$

$$g_{5}(X)=\frac{\sqrt{{\left(745x_{4}/\left(x_{2}x_{3}\right)\right)}^{2}+16.9\cdot 10^{6}}}{110x_{6}^{3}}-1\leq 0,$$

$$g_{6}(X)=\frac{\sqrt{{\left(745x_{5}/\left(x_{2}x_{3}\right)\right)}^{2}+157.5\cdot 10^{6}}}{85x_{7}^{3}}-1\leq 0,$$

$$g_{7}(X)=\frac{x_{2}x_{3}}{40}-1\leq 0,$$

$$g_{8}(X)=\frac{5x_{2}}{x_{1}}-1\leq 0,$$

$$g_{9}(X)=\frac{x_{1}}{12x_{2}}-1\leq 0,$$

$$g_{10}(X)=\frac{1.5x_{6}+1.9}{x_{4}}-1\leq 0,$$

$$g_{11}(X)=\frac{1.1x_{7}+1.9}{x_{5}}-1\leq 0;$$

variable range: $2.6\leq x_{1}\leq 3.6$, $0.7\leq x_{2}\leq 0.8$, $x_{3}\in \left\{17,18,\ldots ,28\right\}$, $7.3\leq x_{4}\leq 8.3$, $7.8\leq x_{5}\leq 8.3$, $2.9\leq x_{6}\leq 3.9$, $5\leq x_{7}\leq 5.5$.

**Figure 15:**
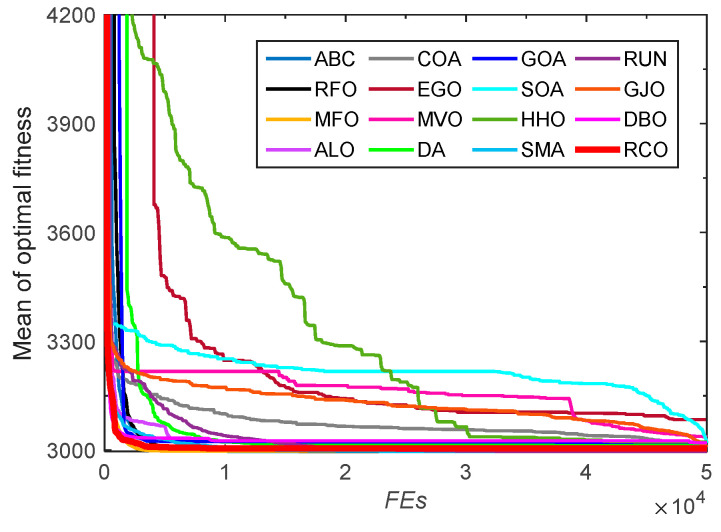
Convergence curves for speed reducer design problem optimized by RCO and other optimizers.

Judging from the statistical results for all optimization algorithms shown in Table 10, RCO, DBO, and MFO together give the best value *F*(*X*) = 2996.34816496 in repeated runs, which is the optimal value of this problem. The corresponding variable solution is *X* = (3.499999999997, 0.7, 17, 7.3, 7.8, 3.350214666096, 5.286683229756). Further, the mean value returned by RCO is not as good as MFO, but the standard deviation is better than MFO. Figure 15 shows the convergence curves for optimizing the speed reducer problem. It can be found that the RCO algorithm can obtain good results with only a small number of function evaluations.

## 5. Conclusions

This paper proposes a new bio-inspired algorithm called Red-crowned Crane Optimization (RCO) algorithm. This algorithm simulates the foraging, roosting, escaping from danger, and crane dance behaviors of the red-crowned crane. The RCO algorithm is tested on the classic CEC-2005 test functions and the newer twelve CEC-2022 test functions. The evaluation results and the signed-rank test results for the benchmark test function set highlight that RCO is highly competitive compared to its competitors. The convergence results show that RCO has a faster convergence speed, and the scalability test results show that RCO can cope with complex high-dimensional tasks. In addition, the sensitivity analysis results reveal that RCO has greater sensitivity to the probability coefficient. The smaller the probability coefficient, the better the exploitation performance of RCO, and the larger the probability coefficient, the better the exploration performance of RCO. The statistical and signed-rank test results for the CEC-2022 function set also demonstrate that RCO is effective and has certain advantages. The RCO algorithm is also used to solve eight reported classic engineering problems to verify its ability to cope with problems with constraints and discrete variables. The optimization results show that RCO is able to provide highly competitive solutions to these problems. Therefore, the RCO algorithm can be used as an alternative optimization algorithm.

In this study, the algorithm uses a certain number of iterations or function evaluations as the termination condition. In future research, to save computational costs, we will attempt other criteria, such as changes in the objective function and the accuracy of optimization results. More importantly, we plan to apply this algorithm to solve controller parameter optimization and robot path planning problems involved in our field.

## Figures and Tables

**Table 1:** A summary of metaheuristic algorithms.

Type	Algorithm	Inspiration
Evolution-based	Genetic Algorithm (GA) [9]	Mutation, crossover, and natural selection strategies
	Genetic Programming (GP) [31]	Inherited the basic idea of GA
	Differential Evolution (DE) [10]	Inherited the basic idea of GA
Swarm-based	Particle Swarm Optimization (PSO) [11]	Predation behavior of birds
	Grey Wolf Optimizer (GWO) [14]	Hierarchy and hunting behavior of gray wolves
	Moth–Flame Optimization (MFO) [32]	Navigation method of moths
	Harris Hawks Optimizer (HHO) [33]	Cooperative and chasing behaviors of Harris’ hawks
	Dung Beetle Optimizer (DBO) [34]	Five behaviors of dung beetles
	Mantis Search Algorithm (MSA) [35]	Hunting and sexual cannibalism of praying mantises
Human-based	Teaching–Learning-Based Optimization (TLBO) [18]	Impact of teachers on student learning
	Student Psychology-Based Optimization (SPBO) [19]	Psychology of students expecting for progress
	Social Network Search (SNS) [36]	Interactive behavior among users in social networks
Physics- and chemistry-based	Simulated Annealing (SA) [37]	Annealing process in physics
	Gravitational Search Algorithm (GSA) [23]	Newton’s law of universal gravitation
	Multi-Verse Optimizer (MVO) [24]	Concepts of white hole, black hole, and wormhole
Others	Sine–Cosine Algorithm (SCA) [27]	Mathematical model of sine and cosine functions
	Arithmetic Optimization Algorithm (AOA) [28]	Main arithmetic operators in mathematics
	Weighted Mean of Vectors (INFO) [38]	Idea of weighted mean

**Table 2:** Parameter settings for all algorithms, where the parameters of RCO are set based on the need to balance exploration and exploitation and the parameter settings of other algorithms follow the recommendations in their original text.

Algorithm	Parameter Settings
RCO	*p_c_* = 0.7, *k*:(*n* − *k*) = 1:1
DBO	*k* = *λ* = 0.1, *b* = 0.3, *S* = 0.5
GJO	*c*_1_ = 1.5
RUN	*a* = 20, *b* = 12
SMA	*v_c_* = 1 − *t*/*t*_max_, *z* = 0.03
HHO	*E*_0_ randomly changes in (−1,1)
COA	*I* randomly changes in {1,2}
EGO	*a* = 2 − 2*t/*t*_max_
RFO	*β* = 0.000001, *e*_0_ = 1, *e*_1_ = 3, *c*_0_ = 2, *c*_1_ = 2, *a*_0_ = 2, *a*_1_ = 3

**Table 3:** Wilcoxon signed-rank test results for CEC-2005 functions.

	Index	RCO vs. DBO	RCO vs. GJO	RCO vs. RUN	RCO vs. SMA	RCO vs. HHO	RCO vs. COA	RCO vs. EGO	RCO vs. RFO
*F*_1_	*p*-value	2.5631 × 10^−6^	1.7344 × 10^−6^	1.7344 × 10^−6^	1	1.7344 × 10^−6^	1.7344 × 10^−6^	1	1.7344 × 10^−6^
	R+	0	0	0	0	0	0	0	0
	R−	435	465	465	0	465	465	0	465
	+/=/−	+	+	+	=	+	+	=	+
*F*_2_	*p*-value	1.7344 × 10^−6^	1.7344 × 10^−6^	1.7344 × 10^−6^	2.1336 × 10^−1^	1.7344 × 10^−6^	1.7344 × 10^−6^	1.7344 × 10^−6^	1.7344 × 10^−6^
	R+	0	0	0	172	0	0	0	0
	R−	465	465	465	293	465	465	465	465
	+/=/−	+	+	+	=	+	+	+	+
*F*_3_	*p*-value	1.7344 × 10^−6^	1.7344 × 10^−6^	1.7344 × 10^−6^	1	1.7344 × 10^−6^	1.7344 × 10^−6^	1	1.7344 × 10^−6^
	R+	0	0	0	0	0	0	0	0
	R−	465	465	465	0	465	465	0	465
	+/=/−	+	+	+	=	+	+	=	+
*F*_4_	*p*-value	1.7344 × 10^−6^	1.7344 × 10^−6^	1.7344 × 10^−6^	1.2544 × 10^−1^	1.7344 × 10^−6^	1.7344 × 10^−6^	1.7344 × 10^−6^	1.7344 × 10^−6^
	R+	0	0	0	307	0	0	0	0
	R−	465	465	465	158	465	465	465	465
	+/=/−	+	+	+	=	+	+	+	+
*F*_5_	*p*-value	1.7344 × 10^−6^	1.7344 × 10^−6^	3.1849 × 10^−1^	1.7344 × 10^−6^	1.7344 × 10^−6^	1.7344 × 10^−6^	1.7344 × 10^−6^	1.9209 × 10^−6^
	R+	0	0	281	465	465	465	0	464
	R−	465	465	184	0	0	0	465	1
	+/=/−	+	+	=	−	−	−	+	−
*F*_6_	*p*-value	1.7344 × 10^−6^	1.7344 × 10^−6^	1.7344 × 10^−6^	1.7344 × 10^−6^	3.8822 × 10^−6^	1.7344 × 10^−6^	1.7344 × 10^−6^	1.7344 × 10^−6^
	R+	465	0	465	0	8	0	0	0
	R−	0	465	0	465	457	465	465	465
	+/=/−	−	+	−	+	+	+	+	+
*F*_7_	*p*-value	1.7344 × 10^−6^	1.9646 × 10^−3^	1.3601 × 10^−5^	1.8326 × 10^−3^	2.2102 × 10^−1^	5.8571 × 10^−1^	3.2857 × 10^−1^	1.7344 × 10^−6^
	R+	0	82	21	81	173	259	280	0
	R−	465	383	444	384	292	206	185	465
	+/=/−	+	+	+	+	=	=	=	+
*F*_8_	*p*-value	1.7988 × 10^−5^	1.7344 × 10^−6^	9.2710 × 10^−3^	1.7344 × 10^−6^	1.7344 × 10^−6^	1.7344 × 10^−6^	8.9443 × 10^−4^	4.4463 × 10^−2^
	R+	441	0	359	465	465	465	71	139
	R−	24	465	106	0	0	0	394	326
	+/=/−	−	+	−	−	−	−	+	+
*F*_9_	*p*-value	1	1	1	1	1	1	1	3.1250 × 10^−2^
	R+	0	0	0	0	0	0	0	0
	R−	0	0	0	0	0	0	0	21
	+/=/−	=	=	=	=	=	=	=	+
*F*_10_	*p*-value	1	1.0135 × 10^−7^	1	1	1	1	1	1
	R+	0	0	0	0	0	0	0	0
	R−	0	465	0	0	0	0	0	0
	+/=/−	=	+	=	=	=	=	=	=
*F*_11_	*p*-value	1	1	1	1	1	1	1	1.2500 × 10^−1^
	R+	0	0	0	0	0	0	0	0
	R−	0	0	0	0	0	0	0	10
	+/=/−	=	=	=	=	=	=	=	=
*F*_12_	*p*-value	3.1123 × 10^−5^	1.7344 × 10^−6^	1.7344 × 10^−6^	1.7344 × 10^−6^	2.7653 × 10^−3^	1.7344 × 10^−6^	1.7344 × 10^−6^	1.2544 × 10^−1^
	R+	435	0	465	0	87	0	0	158
	R−	30	465	0	465	378	465	465	307
	+/=/−	−	+	−	+	+	+	+	=
*F*_13_	*p*-value	4.2857 × 10^−6^	1.7088 × 10^−3^	2.1266 × 10^−6^	1.9209 × 10^−6^	1.7344 × 10^−6^	1.7344 × 10^−6^	6.7328 × 10^−1^	1.7344 × 10^−6^
	R+	456	80	463	464	465	465	253	465
	R−	9	385	2	1	0	0	212	0
	+/=/−	−	+	−	−	−	−	=	−
*F*_14_	*p*-value	1.0881 × 10^−1^	1.6678 × 10^−6^	4.7045 × 10^−4^	2.4730 × 10^−6^	2.5631 × 10^−6^	5.6061 × 10^−6^	1.7344 × 10^−6^	1
	R+	0	0	0	0	0	0	0	0
	R−	6	465	120	435	435	378	465	0
	+/=/−	=	+	+	+	+	+	+	=
*F*_15_	*p*-value	2.0515 × 10^−4^	1.1093 × 10^−1^	1.9569 × 10^−2^	1.8326 × 10^−3^	4.9498 × 10^−2^	1.3601 × 10^−5^	4.1955 × 10^−4^	6.7328 × 10^−1^
	R+	52	310	119	81	137	21	61	253
	R−	413	155	346	384	328	444	404	212
	+/=/−	+	=	+	+	+	+	+	=
*F*_16_	*p*-value	1	1.7344 × 10^−6^	3.8710 × 10^−5^	1.7279 × 10^−6^	7.6227 × 10^−4^	2.5631 × 10^−6^	1.7344 × 10^−6^	1
	R+	0	0	0	0	0	0	0	0
	R−	0	465	253	465	105	435	465	0
	+/=/−	=	+	+	+	+	+	+	=
*F*_17_	*p*-value	1	1.7344 × 10^−6^	2.6414 × 10^−5^	1.7344 × 10^−6^	8.2981 × 10^−6^	1.7344 × 10^−6^	1.7344 × 10^−6^	1
	R+	0	0	0	0	0	0	0	0
	R−	0	465	276	465	351	465	465	0
	+/=/−	=	+	+	+	+	+	+	=
*F*_18_	*p*-value	2.8557 × 10^−5^	1.7344 × 10^−6^	1.8072 × 10^−5^	1.7257 × 10^−6^	2.5596 × 10^−6^	1.7344 × 10^−6^	1.7344 × 10^−6^	1
	R+	0	0	0	0	0	0	0	0
	R−	190	465	300	465	435	465	465	0
	+/=/−	+	+	+	+	+	+	+	=
*F*_19_	*p*-value	4.0479 × 10^−2^	1.7344 × 10^−6^	1.7344 × 10^−6^	1.7344 × 10^−6^	1.7344 × 10^−6^	1.7344 × 10^−6^	1.7344 × 10^−6^	1
	R+	1	0	0	0	0	0	0	0
	R−	20	465	465	465	465	465	465	0
	+/=/−	+	+	+	+	+	+	+	=
*F*_20_	*p*-value	3.2082 × 10^−2^	3.1123 × 10^−5^	2.1053 × 10^−3^	1.3601 × 10^−5^	1.6046 × 10^−4^	1.7344 × 10^−6^	4.0702 × 10^−2^	7.2488 × 10^−1^
	R+	36.5	30	83	21	49	0	133	149.5
	R−	134.5	435	382	444	416	465	332	175.5
	+/=/−	+	+	+	+	+	+	+	=
*F*_21_	*p*-value	1.8965 × 10^−4^	1.7344 × 10^−6^	1.7344 × 10^−6^	1.7344 × 10^−6^	1.7344 × 10^−6^	1.7344 × 10^−6^	1.7344 × 10^−6^	9.7656 × 10^−4^
	R+	0	0	0	0	0	0	0	0
	R−	171	465	465	465	465	465	465	66
	+/=/−	+	+	+	+	+	+	+	+
*F*_22_	*p*-value	6.2096 × 10^−4^	1.7344 × 10^−6^	1.7344 × 10^−6^	1.7344 × 10^−6^	1.7344 × 10^−6^	1.7344 × 10^−6^	1.7344 × 10^−6^	1.5625 × 10^−2^
	R+	0	0	0	0	0	0	0	0
	R−	120	465	465	465	465	465	465	28
	+/=/−	+	+	+	+	+	+	+	+
*F*_23_	*p*-value	3.1915 × 10^−3^	1.7344 × 10^−6^	1.7344 × 10^−6^	1.7344 × 10^−6^	1.7344 × 10^−6^	1.7344 × 10^−6^	1.7344 × 10^−6^	1.9531 × 10^−3^
	R+	0	0	0	0	0	0	0	0
	R−	66	465	465	465	465	465	465	55
	+/=/−	+	+	+	+	+	+	+	+
Unimodal (+/=/−)		6/0/1	7/0/0	5/1/1	2/4/1	5/1/1	5/1/1	4/3/0	6/0/1
Multimodal (+/=/−)		7/6/3	13/3/0	10/3/3	11/3/2	11/3/2	11/3/2	12/4/0	5/10/1
Total (+/=/−)		13/6/4	20/3/0	15/4/4	13/7/3	16/4/3	16/4/3	16/7/0	11/10/2

**Table 4:** Evaluation results for scalable functions (*F*_1_–*F*_13_) in 500 dimensions optimized by RCO and other optimizers.

	Index	RCO	DBO	GJO	RUN	SMA	HHO	COA	EGO	RFO
*F*_1_	Mean	0	3.2549 × 10^−234^	4.9056 × 10^−34^	5.1469 × 10^−206^	0	1.0271 × 10^−187^	1.0905 × 10^−300^	0	1.2147 × 10^−116^
	Std	0	1.7826 × 10^−233^	5.4342 × 10^−34^	2.8173 × 10^−205^	0	5.6213 × 10^−187^	5.9725 × 10^−300^	0	6.6469 × 10^−116^
	Min	0	3.7701 × 10^−297^	3.1716 × 10^−35^	2.6183 × 10^−229^	0	3.0699 × 10^−213^	0	0	3.8455 × 10^−146^
	Max	0	9.7636 × 10^−233^	2.1880 × 10^−33^	1.5431 × 10^−204^	0	3.0790 × 10^−186^	3.2713 × 10^−299^	0	3.6408 × 10^−115^
	Rank	1	5	9	6	1	7	4	1	8
*F*_2_	Mean	1.6764 × 10^−231^	8.2119 × 10^−125^	1.1235 × 10^−21^	3.2985 × 10^−117^	1.2861 × 10^−1^	2.8828 × 10^−99^	1.2626 × 10^−151^	9.3039 × 10^−224^	1.9514 × 10^−60^
	Std	9.0402 × 10^−231^	3.6042 × 10^−124^	7.4570 × 10^−22^	1.7917 × 10^−116^	4.5867 × 10^−1^	9.0091 × 10^−99^	5.6229 × 10^−151^	5.0513 × 10^−223^	1.0687 × 10^−59^
	Min	1.8057 × 10^−269^	1.9735 × 10^−151^	3.3215 × 10^−22^	2.9994 × 10^−126^	3.2039 × 10^−62^	9.6944 × 10^−110^	4.0545 × 10^−163^	1.0810 × 10^−233^	2.0457 × 10^−76^
	Max	4.9536 × 10^−230^	1.9349 × 10^−123^	4.4806 × 10^−21^	9.8163 × 10^−116^	2.3614 × 10^0^	4.0818 × 10^−98^	3.0789 × 10^−150^	2.7674 × 10^−222^	5.8533 × 10^−59^
	Rank	1	4	8	5	9	6	3	2	7
*F*_3_	Mean	0	5.5813 × 10^−59^	1.3174 × 10^3^	8.8613 × 10^−170^	2.9198 × 10^−204^	2.2178 × 10^−99^	1.7681 × 10^−300^	0	3.5998 × 10^−33^
	Std	0	3.0570 × 10^−59^	3.9531 × 10^3^	4.8535 × 10^−169^	1.5993 × 10^−203^	1.2147 × 10^−98^	9.6727 × 10^−300^	0	1.7931 × 10^−32^
	Min	0	8.8207 × 10^−256^	2.6831 × 10^−2^	7.1550 × 10^−194^	0	1.7423 × 10^−163^	8.0533 × 10^−322^	0	7.6499 × 10^−62^
	Max	0	1.6744 × 10^−58^	1.9815 × 10^4^	2.6584 × 10^−168^	8.7595 × 10^−203^	6.6534 × 10^−98^	5.2982 × 10^−299^	0	9.8324 × 10^−32^
	Rank	1	7	9	5	4	6	3	1	8
*F*_4_	Mean	1.2991 × 10^−221^	3.1022 × 10^−86^	7.3632 × 10^1^	4.0938 × 10^−98^	1.7504 × 10^−151^	8.2143 × 10^−96^	3.9586 × 10^−153^	9.9172 × 10^−197^	1.1167 × 10^−32^
	Std	7.1135 × 10^−221^	1.3699 × 10^−85^	5.4243 × 10^0^	1.8918 × 10^−97^	9.5872 × 10^−151^	3.3751 × 10^−95^	1.1159 × 10^−152^	3.7978 × 10^−196^	3.5729 × 10^−32^
	Min	1.1104 × 10^−263^	6.2548 × 10^−145^	6.3334 × 10^1^	1.5590 × 10^−111^	0	3.1642 × 10^−107^	1.0136 × 10^−161^	7.2829 × 10^−204^	1.1963 × 10^−43^
	Max	3.8963 × 10^−220^	7.3011 × 10^−85^	8.4766 × 10^1^	1.0228 × 10^−96^	5.2511 × 10^−150^	1.8161 × 10^−94^	4.8629 × 10^−152^	2.0742 × 10^−195^	1.8240 × 10^−31^
	Rank	1	7	9	5	4	6	3	2	8
*F*_5_	Mean	4.9166 × 10^2^	4.9692 × 10^2^	4.9815 × 10^2^	4.9274 × 10^2^	3.8907 × 10^1^	2.5573 × 10^−2^	2.5121 × 10^0^	4.9713 × 10^2^	4.9694 × 10^2^
	Std	4.6246 × 10^−1^	3.8883 × 10^−1^	4.2371 × 10^−1^	1.5641 × 10^0^	7.6452 × 10^1^	2.8135 × 10^−2^	4.2257 × 10^0^	3.1220 × 10^−1^	4.4343 × 10^−1^
	Min	4.9054 × 10^2^	4.9609 × 10^2^	4.9676 × 10^2^	4.8966 × 10^2^	4.5526 × 10^−2^	2.6476 × 10^−5^	5.0683 × 10^−3^	4.9664 × 10^2^	4.9566 × 10^2^
	Max	4.9250 × 10^2^	4.9782 × 10^2^	4.9844 × 10^2^	4.9476 × 10^2^	3.6236 × 10^2^	1.0743 × 10^−1^	1.6977 × 10^1^	4.9774 × 10^2^	4.9746 × 10^2^
	Rank	4	6	9	5	3	1	2	8	7
*F*_6_	Mean	7.5351 × 10^−8^	7.0061 × 10^1^	1.1072 × 10^2^	7.8535 × 10^−1^	5.3724 × 10^0^	2.1118 × 10^−4^	1.7517 × 10^−1^	1.1613 × 10^2^	4.8723 × 10^1^
	Std	9.9886 × 10^−8^	3.4698 × 10^0^	1.4933 × 10^0^	2.2126 × 10^−1^	7.2437 × 10^0^	2.2250 × 10^−4^	2.9082 × 10^−1^	1.6564 × 10^0^	4.6534 × 10^0^
	Min	3.5668 × 10^−9^	6.4904 × 10^1^	1.0658 × 10^2^	3.9644 × 10^−1^	4.2435 × 10^−5^	4.3670 × 10^−8^	1.7219 × 10^−6^	1.1171 × 10^2^	3.8296 × 10^1^
	Max	3.8987 × 10^−7^	7.7117 × 10^1^	1.1384 × 10^2^	1.1747 × 10^0^	3.0000 × 10^1^	9.0233 × 10^−4^	1.5263 × 10^0^	1.1892 × 10^2^	5.7172 × 10^1^
	Rank	1	7	8	4	5	2	3	9	6
*F*_7_	Mean	3.4765 × 10^−5^	5.0432 × 10^−4^	8.2868 × 10^−4^	1.6166 × 10^−4^	3.7354 × 10^−4^	7.6822 × 10^−5^	8.6069 × 10^−5^	2.9107 × 10^−3^	6.7328 × 10^−3^
	Std	3.6087 × 10^−5^	4.4814 × 10^−4^	5.6353 × 10^−4^	1.2099 × 10^−4^	4.3033 × 10^−4^	8.3173 × 10^−5^	7.6387 × 10^−5^	2.8227 × 10^−3^	5.9478 × 10^−3^
	Min	1.3065 × 10^−6^	6.8894 × 10^−5^	2.5234 × 10^−4^	1.7797 × 10^−5^	1.3430 × 10^−5^	5.1595 × 10^−7^	4.7560 × 10^−6^	1.2498 × 10^−4^	6.0707 × 10^−4^
	Max	1.4808 × 10^−4^	2.0633 × 10^−3^	2.4861 × 10^−3^	4.8836 × 10^−4^	1.7325 × 10^−3^	4.2642 × 10^−4^	2.9340 × 10^−4^	1.0039 × 10^−2^	2.7323 × 10^−2^
	Rank	1	6	7	4	5	2	3	8	9
*F*_8_	Mean	−9.9019 × 10^4^	−1.8160 × 10^5^	−3.3607 × 10^4^	−9.4850 × 10^4^	−2.0948 × 10^5^	−2.0949 × 10^5^	−2.0949 × 10^5^	−1.0313 × 10^5^	−9.6555 × 10^4^
	Std	1.0804 × 10^4^	1.1723 × 10^4^	1.7736 × 10^4^	1.6511 × 10^4^	2.1871 × 10^1^	1.5231 × 10^0^	6.0066 × 10^−1^	1.2180 × 10^3^	6.9129 × 10^3^
	Min	−1.2597 × 10^5^	−1.9880 × 10^5^	−7.1206 × 10^4^	−1.2633 × 10^5^	−2.0949 × 10^5^	−2.0949 × 10^5^	−2.0949 × 10^5^	−1.0654 × 10^5^	−1.1127 × 10^5^
	Max	−8.1295 × 10^4^	−1.5141 × 10^5^	−1.1479 × 10^4^	−6.2180 × 10^4^	−2.0937 × 10^5^	−2.0949 × 10^5^	−2.0949 × 10^5^	−1.0169 × 10^5^	−8.3802 × 10^4^
	Rank	6	4	9	8	3	2	1	5	7
*F*_9_	Mean	0	0	0	0	0	0	0	0	3.1686 × 10^−1^
	Std	0	0	0	0	0	0	0	0	1.7355 × 10^0^
	Min	0	0	0	0	0	0	0	0	0
	Max	0	0	0	0	0	0	0	0	9.5059 × 10^0^
	Rank	1	1	1	1	1	1	1	1	9
*F*_10_	Mean	8.8818 × 10^−16^	1.0066 × 10^−15^	3.4521 × 10^−14^	8.8818 × 10^−16^	8.8818 × 10^−16^	8.8818 × 10^−16^	8.8818 × 10^−16^	8.8818 × 10^−16^	8.8818 × 10^−16^
	Std	0	6.4863 × 10^−16^	4.1445 × 10^−15^	0	0	0	0	0	0
	Min	8.8818 × 10^−16^	8.8818 × 10^−16^	2.9310 × 10^−14^	8.8818 × 10^−16^	8.8818 × 10^−16^	8.8818 × 10^−16^	8.8818 × 10^−16^	8.8818 × 10^−16^	8.8818 × 10^−16^
	Max	8.8818 × 10^−16^	4.4409 × 10^−15^	3.9968 × 10^−14^	8.8818 × 10^−16^	8.8818 × 10^−16^	8.8818 × 10^−16^	8.8818 × 10^−16^	8.8818 × 10^−16^	8.8818 × 10^−16^
	Rank	1	8	9	1	1	1	1	1	1
*F*_11_	Mean	0	0	8.1416 × 10^−17^	0	0	0	0	0	5.1196 × 10^−4^
	Std	0	0	4.9935 × 10^−17^	0	0	0	0	0	2.8041 × 10^−3^
	Min	0	0	0	0	0	0	0	0	0
	Max	0	0	1.1102 × 10^−16^	0	0	0	0	0	1.5359 × 10^−2^
	Rank	1	1	8	1	1	1	1	1	9
*F*_12_	Mean	5.1880 × 10^−7^	3.8002 × 10^−1^	9.3776 × 10^−1^	9.9025 × 10^−4^	3.2460 × 10^−4^	6.6731 × 10^−7^	4.8266 × 10^−5^	8.8587 × 10^−1^	1.8152 × 10^−1^
	Std	1.4180 × 10^−7^	3.4013 × 10^−2^	2.8596 × 10^−2^	1.2189 × 10^−3^	3.9289 × 10^−4^	6.9197 × 10^−7^	7.5522 × 10^−5^	1.0236 × 10^−1^	3.8451 × 10^−2^
	Min	7.7506 × 10^−10^	2.9454 × 10^−1^	8.8196 × 10^−1^	5.4244 × 10^−4^	1.6356 × 10^−7^	2.5876 × 10^−9^	3.5303 × 10^−7^	5.2520 × 10^−1^	1.2009 × 10^−1^
	Max	7.3749 × 10^−7^	4.3693 × 10^−1^	9.9715 × 10^−1^	7.3945 × 10^−3^	1.2682 × 10^−3^	8.4475 × 10^−7^	3.3038 × 10^−4^	1.0450 × 10^0^	2.5459 × 10^−1^
	Rank	1	7	9	5	4	2	3	8	6
*F*_13_	Mean	8.3503 × 10^−1^	4.8909 × 10^1^	4.8170 × 10^1^	2.1474 × 10^0^	1.9215 × 10^−1^	4.8130 × 10^−5^	4.4956 × 10^−3^	4.5826 × 10^1^	4.7719 × 10^1^
	Std	5.9713 × 10^−1^	2.6817 × 10^−1^	3.9525 × 10^−1^	6.8741 × 10^−1^	3.8994 × 10^−1^	4.9497 × 10^−5^	7.2180 × 10^−3^	4.5337 × 10^0^	2.0191 × 10^0^
	Min	2.9012 × 10^−7^	4.8491 × 10^1^	4.7390 × 10^1^	1.2182 × 10^0^	5.1525 × 10^−5^	3.7797 × 10^−7^	4.3281 × 10^−5^	3.3137 × 10^1^	4.2382 × 10^1^
	Max	1.7359 × 10^0^	4.9563 × 10^1^	4.8971 × 10^1^	3.8496 × 10^0^	1.7122 × 10^0^	1.5601 × 10^−4^	3.4253 × 10^−2^	4.9384 × 10^1^	4.9637 × 10^1^
	Rank	4	9	8	5	3	1	2	6	7
Mean rank		2.6923	6.0385	8.1923	4.9615	4.1923	3.6538	3.0385	4.9231	7.3077
Total rank		1	7	9	6	4	3	2	5	8

**Table 5:** Evaluation results for benchmark test functions optimized by RCO in the case of different probability coefficients.

	Index	0.1	0.3	0.5	0.7	0.9
*F*_1_	Mean	0	0	0	0	5.5895 × 10^−146^
	Std	0	0	0	0	3.0615 × 10^−145^
*F*_2_	Mean	0	0	0	1.9434 × 10^−238^	1.0826 × 10^−78^
	Std	0	0	0	1.0645 × 10^−237^	5.7653 × 10^−78^
*F*_3_	Mean	0	0	0	0	9.0527 × 10^−135^
	Std	0	0	0	0	4.9584 × 10^−134^
*F*_4_	Mean	0	0	0	1.3051 × 10^−226^	2.4421 × 10^−73^
	Std	0	0	0	7.1478 × 10^−226^	9.8518 × 10^−73^
*F*_5_	Mean	2.3727 × 10^1^	2.3416 × 10^1^	2.3906 × 10^1^	2.3255 × 10^1^	2.4518 × 10^1^
	Std	5.5787 × 10^−1^	2.4010 × 10^−1^	4.0693 × 10^−1^	1.3112 × 10^−1^	2.6634 × 10^−1^
*F*_6_	Mean	9.7394 × 10^−1^	7.5580 × 10^−3^	1.9383 × 10^−5^	8.2703 × 10^−8^	7.6147 × 10^−9^
	Std	4.4785 × 10^−1^	1.6890 × 10^−2^	2.4897 × 10^−5^	8.8799 × 10^−8^	1.5297 × 10^−8^
*F*_7_	Mean	2.2990 × 10^−5^	1.7420 × 10^−5^	4.2269 × 10^−5^	3.8235 × 10^−5^	1.8210 × 10^−4^
	Std	1.9088 × 10^−5^	2.0308 × 10^−5^	3.8226 × 10^−5^	5.4596 × 10^−5^	2.6879 × 10^−4^
*F*_8_	Mean	−7.7980 × 10^3^	−7.8777 × 10^3^	−7.9839 × 10^3^	−7.8058 × 10^3^	−8.0328 × 10^3^
	Std	1.2775 × 10^3^	1.3528 × 10^3^	1.0981 × 10^3^	1.1148 × 10^3^	1.0466 × 10^3^
*F*_9_	Mean	0	0	0	0	0
	Std	0	0	0	0	0
*F*_10_	Mean	8.8818 × 10^−16^	8.8818 × 10^−16^	8.8818 × 10^−16^	8.8818 × 10^−16^	8.8818 × 10^−16^
	Std	0	0	0	0	0
*F*_11_	Mean	0	0	0	0	0
	Std	0	0	0	0	0
*F*_12_	Mean	8.2255 × 10^−2^	3.0265 × 10^−2^	9.3620 × 10^−4^	2.1603 × 10^−7^	8.1993 × 10^−8^
	Std	3.4341 × 10^−2^	2.5054 × 10^−2^	1.8180 × 10^−3^	4.7274 × 10^−7^	2.3867 × 10^−7^
*F*_13_	Mean	2.1561 × 10^0^	1.4643 × 10^0^	1.1598 × 10^0^	9.7940 × 10^−1^	9.2470 × 10^−1^
	Std	5.5240 × 10^−1^	4.8535 × 10^−1^	6.4637 × 10^−1^	7.1097 × 10^−1^	7.4855 × 10^−1^
*F*_14_	Mean	1.6924 × 10^0^	1.0458 × 10^0^	9.9800 × 10^−1^	9.9800 × 10^−1^	9.9800 × 10^−1^
	Std	9.7957 × 10^−1^	2.6199 × 10^−1^	4.6963 × 10^−13^	2.3142 × 10^−16^	2.2204 × 10^−16^
*F*_15_	Mean	3.7399 × 10^−4^	3.2535 × 10^−4^	3.1835 × 10^−4^	3.2153 × 10^−4^	3.9879 × 10^−4^
	Std	1.6935 × 10^−4^	6.5432 × 10^−5^	5.4844 × 10^−5^	3.5851 × 10^−5^	1.9682 × 10^−4^
*F*_16_	Mean	−1.0316 × 10^0^	−1.0316 × 10^0^	−1.0316 × 10^0^	−1.0316 × 10^0^	−1.0316 × 10^0^
	Std	1.9433 × 10^−7^	5.1881 × 10^−11^	4.3300 × 10^−15^	5.3761 × 10^−16^	5.2156 × 10^−16^
*F*_17_	Mean	3.9789 × 10^−1^	3.9789 × 10^−1^	3.9789 × 10^−1^	3.9789 × 10^−1^	3.9789 × 10^−1^
	Std	2.5819 × 10^−6^	4.1858 × 10^−10^	1.0725 × 10^−13^	0	0
*F*_18_	Mean	3.0001 × 10^0^	3.0000 × 10^0^	3.0000 × 10^0^	3.0000 × 10^0^	3.0000 × 10^0^
	Std	2.2073 × 10^−4^	2.0626 × 10^−8^	6.2552 × 10^−12^	2.0534 × 10^−15^	2.6453 × 10^−15^
*F*_19_	Mean	−3.8619 × 10^0^	−3.8628 × 10^0^	−3.8628 × 10^0^	−3.8628 × 10^0^	−3.8628 × 10^0^
	Std	1.5473 × 10^−3^	9.0922 × 10^−7^	9.9934 × 10^−13^	2.2494 × 10^−15^	2.4057 × 10^−15^
*F*_20_	Mean	−3.2561 × 10^0^	−3.2630 × 10^0^	−3.2784 × 10^0^	−3.2863 × 10^0^	−3.2943 × 10^0^
	Std	7.4010 × 10^−2^	6.5231 × 10^−2^	5.8281 × 10^−2^	5.5415 × 10^−2^	5.1146 × 10^−2^
*F*_21_	Mean	−1.0151 × 10^1^	−1.0153 × 10^1^	−1.0153 × 10^1^	−1.0153 × 10^1^	−1.0153 × 10^1^
	Std	2.4943 × 10^−3^	3.3099 × 10^−6^	1.3447 × 10^−9^	6.1269 × 10^−15^	5.2051 × 10^−15^
*F*_22_	Mean	−1.0400 × 10^1^	−1.0403 × 10^1^	−1.0403 × 10^1^	−1.0403 × 10^1^	−1.0403 × 10^1^
	Std	3.6378 × 10^−3^	1.8140 × 10^−5^	2.0135 × 10^−9^	2.4240 × 10^−15^	1.3995 × 10^−15^
*F*_23_	Mean	−1.0533 × 10^1^	−1.0536 × 10^1^	−1.0536 × 10^1^	−1.0536 × 10^1^	−1.0536 × 10^1^
	Std	3.7565 × 10^−3^	1.2283 × 10^−5^	1.2123 × 10^−9^	4.6181 × 10^−15^	1.7455 × 10^−15^

**Table 6:** Wilcoxon signed-rank test results for CEC-2022 functions.

	Index	RCO vs. DBO	RCO vs. GJO	RCO vs. RUN	RCO vs. SMA	RCO vs. HHO	RCO vs. COA	RCO vs. EGO	RCO vs. RFO
*F*_24_	*p*-value	7.0356 × 10^−1^	1.7344 × 10^−6^	1.7344 × 10^−6^	1.7344 × 10^−6^	1.7344 × 10^−6^	1.7344 × 10^−6^	1.7344 × 10^−6^	1.7344 × 10^−6^
	R+	251	0	0	0	0	0	0	0
	R−	214	465	465	465	465	465	465	465
	+/=/−	=	+	+	+	+	+	+	+
*F*_2_5__	*p*-value	1.3238 × 10^−3^	2.1630 × 10^−5^	8.9364 × 10^−1^	8.7297 × 10^−3^	1.3591 × 10^−1^	4.6818 × 10^−3^	2.0515 × 10^−4^	2.2985 × 10^−1^
	R+	62	26	226	105	160	95	52	108
	R−	344	439	239	360	305	370	413	192
	+/=/−	+	+	=	+	=	+	+	=
*F*_2_6__	*p*-value	4.4919 × 10^−2^	2.3534 × 10^−6^	8.9364 × 10^−1^	1.7344 × 10^−6^	6.3198 × 10^−5^	9.4261 × 10^−1^	3.8203 × 10^−1^	8.4661 × 10^−6^
	R+	135	3	226	465	38	229	190	449
	R−	330	462	239	0	427	236	275	16
	+/=/−	+	+	=	−	+	=	=	−
*F*_2_7__	*p*-value	2.6229 × 10^−1^	3.1849 × 10^−1^	9.2626 × 10^−1^	8.9364 × 10^−1^	3.4935 × 10^−1^	2.8786 × 10^−6^	9.1694 × 10^−3^	9.1662 × 10^−2^
	R+	178	184	228	226	187	5	97	150.5
	R−	287	281	237	239	278	460	338	314.5
	+/=/−	=	=	=	=	=	+	+	=
*F*_2_8__	*p*-value	7.8647 × 10^−2^	6.1564 × 10^−4^	1.1561 × 10^−1^	1.9209 × 10^−6^	6.3391 × 10^−6^	2.7029 × 10^−2^	6.8836 × 10^−1^	6.9575 × 10^−2^
	R+	147	66	156	464	13	125	252	277
	R−	318	399	309	1	452	340	213	188
	+/=/−	=	+	+	−	+	+	=	=
*F*_2_9__	*p*-value	6.8359 × 10^−3^	3.8822 × 10^−6^	8.6121 × 10^−1^	1.4773 × 10^−4^	7.3433 × 10^−1^	2.1336 × 10^−1^	8.8203 × 10^−3^	9.6266 × 10^−4^
	R+	101	8	241	48	216	172	90	72
	R−	364	457	224	417	249	293	375	393
	+/=/−	+	+	=	+	=	=	+	+
*F*_30_	*p*-value	1.5886 × 10^−1^	3.3886 × 10^−1^	6.4352 × 10^−1^	4.7292 × 10^−6^	1.0639 × 10^−1^	9.9179 × 10^−1^	7.7309 × 10^−3^	6.3391 × 10^−6^
	R+	164	186	210	455	154	232	103	452
	R−	301	279	255	10	311	233	362	13
	+/=/−	=	=	=	−	=	=	+	−
*F*_31_	*p*-value	5.8571 × 10^−1^	6.5641 × 10^−2^	1.4773 × 10^−4^	3.4053 × 10^−5^	9.4261 × 10^−1^	2.8308 × 10^−4^	8.1302 × 10^−1^	5.3197 × 10^−3^
	R+	259	322	417	434	236	56	244	97
	R−	206	143	48	31	229	409	221	368
	+/=/−	=	=	−	−	=	+	=	+
*F*_32_	*p*-value	8.8561 × 10^−4^	1.7344 × 10^−6^	1.7344 × 10^−6^	1.7344 × 10^−6^	1.7344 × 10^−6^	1.7344 × 10^−6^	1.7344 × 10^−6^	1.7344 × 10^−6^
	R+	0	0	0	0	0	0	0	0
	R−	105	465	465	465	465	465	465	465
	+/=/−	+	+	+	+	+	+	+	+
*F*_33_	*p*-value	1.3595 × 10^−4^	1.8910 × 10^−4^	1.4936 × 10^−5^	4.5336 × 10^−4^	6.6392 × 10^−4^	1.7344 × 10^−6^	1.7344 × 10^−6^	1.7344 × 10^−6^
	R+	47	51	22	62	67	0	0	0
	R−	418	414	443	403	398	465	465	465
	+/=/−	+	+	+	+	+	+	+	+
*F*_34_	*p*-value	6.5833 × 10^−1^	9.6266 × 10^−4^	8.2901 × 10^−1^	4.9080 × 10^−1^	2.0589 × 10^−1^	6.4242 × 10^−3^	2.4308 × 10^−2^	5.7517 × 10^−6^
	R+	254	72	222	199	171	100	123	12
	R−	211	393	243	266	294	365	342	453
	+/=/−	=	+	=	=	=	+	+	+
*F*_35_	*p*-value	3.3269 × 10^−2^	4.0483 × 10^−1^	2.5967 × 10^−5^	1.9209 × 10^−6^	7.6909 × 10^−6^	1.7344 × 10^−6^	1.9209 × 10^−6^	1.1265 × 10^−5^
	R+	129	192	437	464	15	0	1	19
	R−	336	273	28	1	450	465	464	446
	+/=/−	+	=	−	−	+	+	+	+
Total (+/=/−)		6/6/0	8/4/0	4/6/2	5/2/5	6/6/0	9/3/0	9/3/0	7/3/2

**Table 7:** Evaluation results for three-bar truss design problem optimized by RCO and other optimizers.

Optimizer	Mean	Std	Best	Worst
RCO	263.89756856	2.8392 × 10^−3^	263.89584466	263.90810098
DBO	263.89599408	1.6833 × 10^−4^	263.89584349	263.89636188
GJO	263.90281430	4.7808 × 10^−3^	263.89653293	263.91402442
RUN	263.90804643	3.6576 × 10^−2^	263.89585913	264.09314182
SMA	269.04243746	2.3510 × 10^0^	264.27175200	272.76121135
HHO	263.93731983	6.0537 × 10^−2^	263.89585959	264.15000833
SOA	265.18797575	4.7991 × 10^0^	263.89778678	282.84271247
GOA	263.94396581	8.8377 × 10^−2^	263.89585342	264.20396976
DA	263.90640435	1.4283 × 10^−2^	263.89597194	263.95468591
MVO	263.90726865	1.5644 × 10^−3^	263.89586605	263.91319598
EGO	264.08615778	1.6593 × 10^−1^	263.92084673	264.72395877
COA	264.00727860	1.4136 × 10^−1^	263.89594398	264.47160208
ALO	263.90617001	4.1912 × 10^−4^	263.89586378	263.90800028
MFO	263.91766697	2.7763 × 10^−2^	263.89589229	263.99424724
RFO	263.91886530	3.4853 × 10^−2^	263.89584948	264.06851817
ABC	263.90089934	3.3974 × 10^−3^	263.89660081	263.90982347

**Table 8:** Evaluation results for cantilever beam design problem optimized by RCO and other optimizers.

Optimizer	Mean	Std	Best	Worst
RCO	1.34000633	2.2786 × 10^−5^	1.33995802	1.34009808
DBO	1.33995946	2.7255 × 10^−6^	1.33995667	1.33996851
GJO	1.34008587	7.1985 × 10^−5^	1.33998254	1.34029105
RUN	1.33995971	2.7643 × 10^−6^	1.33995681	1.33996632
SMA	1.34009064	1.0247 × 10^−4^	1.33997213	1.34038478
HHO	1.34198908	1.2892 × 10^−3^	1.34028452	1.34471428
SOA	1.34060442	3.6542 × 10^−4^	1.34006455	1.34150095
GOA	1.34180335	4.0402 × 10^−3^	1.33997592	1.36152295
DA	1.34939886	6.3933 × 10^−3^	1.34041507	1.36246818
MVO	1.34044801	2.9033 × 10^−4^	1.34004248	1.34140214
EGO	1.35278100	5.8881 × 10^−3^	1.34397013	1.36625739
COA	1.44514835	4.6579 × 10^−2^	1.36208825	1.52706561
ALO	1.34001504	5.6375 × 10^−5^	1.33996297	1.34025313
MFO	1.34026388	2.5869 × 10^−4^	1.33996126	1.34089458
RFO	1.34106020	2.6982 × 10^−3^	1.33995882	1.35122774
ABC	1.34021861	9.4754 × 10^−5^	1.34005992	1.34050604

**Table 9:** Evaluation results for corrugated bulkhead design problem optimized by RCO and other optimizers.

Optimizer	Mean	Std	Best	Worst
RCO	6.84634282	7.9957 × 10^−3^	6.84295801	6.88137318
DBO	6.85371900	5.8940 × 10^−2^	6.84295801	7.16578788
GJO	6.95430310	3.5572 × 10^−1^	6.84580979	8.26480456
RUN	6.86424400	1.1607 × 10^−1^	6.84297576	7.47881321
SMA	11.56046091	1.2513 × 10^0^	8.18353269	12.45148765
HHO	7.04656180	1.8495 × 10^−1^	6.85776424	7.53023436
SOA	7.59063457	6.9362 × 10^−1^	6.85965671	8.28438401
GOA	8.07162333	4.7083 × 10^−1^	7.03459838	8.80221181
DA	7.01936369	6.2798 × 10^−1^	6.84420883	10.31687564
MVO	6.85330642	7.1921 × 10^−3^	6.84378706	6.86921410
EGO	7.14424015	3.1709 × 10^−1^	6.95086094	8.54195460
COA	7.44862057	2.6788 × 10^−1^	7.00682703	8.25761889
ALO	6.91410825	9.1423 × 10^−2^	6.84296760	7.28100558
MFO	6.84295811	8.7974 × 10^−7^	6.84295801	6.84296066
RFO	6.84341532	1.7596 × 10^−3^	6.84295801	6.85264001
ABC	6.84323956	1.6049 × 10^−4^	6.84298332	6.84363911

**Table 10:** Evaluation results for speed reducer design problem optimized by RCO and other optimizers.

Optimizer	Mean	Std	Best	Worst
RCO	3004.77871743	6.3584 × 10^0^	2996.34816496	3016.62134716
DBO	3019.69646856	3.6985 × 10^1^	2996.34816496	3188.32450766
GJO	3008.29523532	4.5195 × 10^0^	3002.65647414	3018.77341248
RUN	2996.36244665	1.2103 × 10^−2^	2996.35052416	2996.40167482
SMA	2996.34842012	3.1748 × 10^−4^	2996.34819234	2996.34996928
HHO	3022.70732094	1.1816 × 10^1^	3004.68594421	3055.73175253
SOA	3020.70262569	1.0990 × 10^1^	3006.32501492	3050.35832119
GOA	3021.58587579	1.5643 × 10^1^	3010.78997040	3039.52619342
DA	3015.08718301	1.7095 × 10^1^	2998.16375095	3056.04561087
MVO	3036.79926778	1.8310 × 10^1^	3011.09568733	3086.79861945
EGO	3084.75407037	4.5309 × 10^1^	3039.50902406	3197.67885658
COA	3009.29775834	2.8429 × 10^0^	2998.06942756	3030.25654118
ALO	3003.41214141	5.0234 × 10^0^	2996.35200867	3014.25227204
MFO	2997.96853997	7.3133 × 10^0^	2996.34816496	3035.62557865
RFO	2996.75478571	1.9620 × 10^0^	2996.34816501	3007.10936746
ABC	2996.36592281	7.7803 × 10^−3^	2996.35437282	2996.38186476

**Figure A1:**
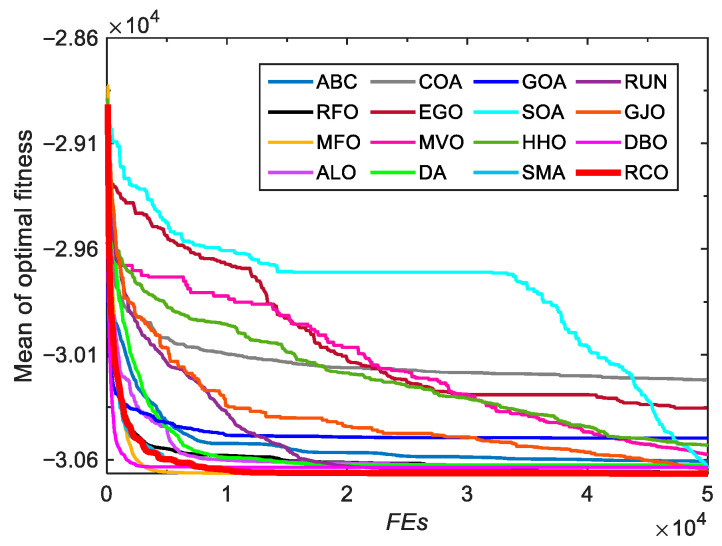
Convergence curves for Himmelblau’s nonlinear problem optimized by RCO and other optimizers.

**Figure A2:**
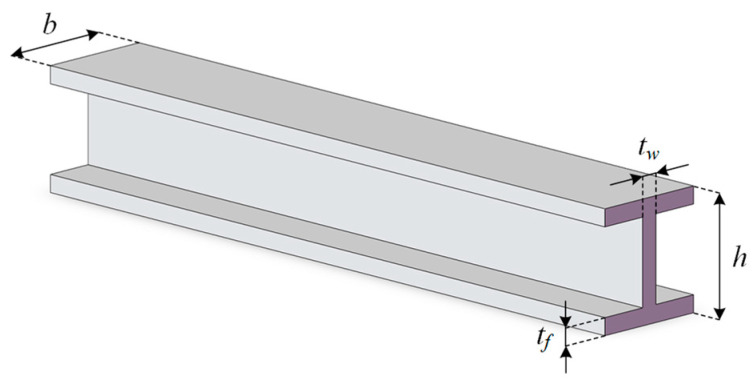
Structure diagram of I-beam.

**Figure A3:**
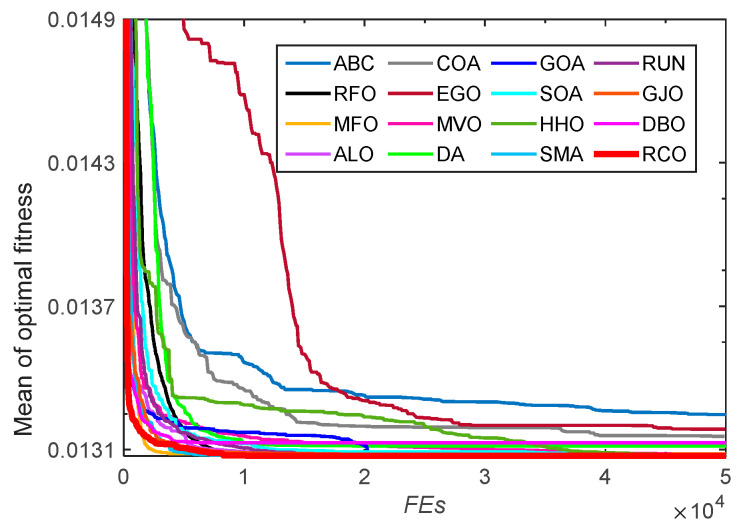
Convergence curves for I-beam design problem optimized by RCO and other optimizers.

**Figure A4:**
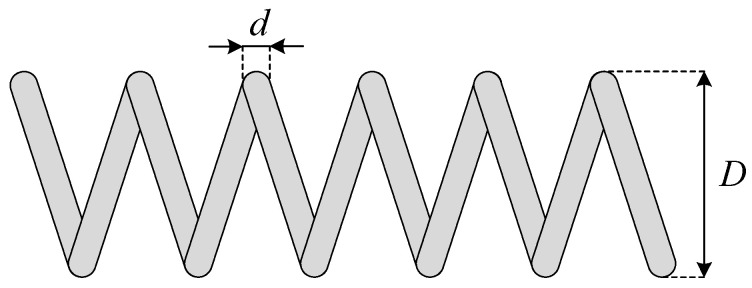
Structure diagram of tension/compression spring.

**Figure A5:**
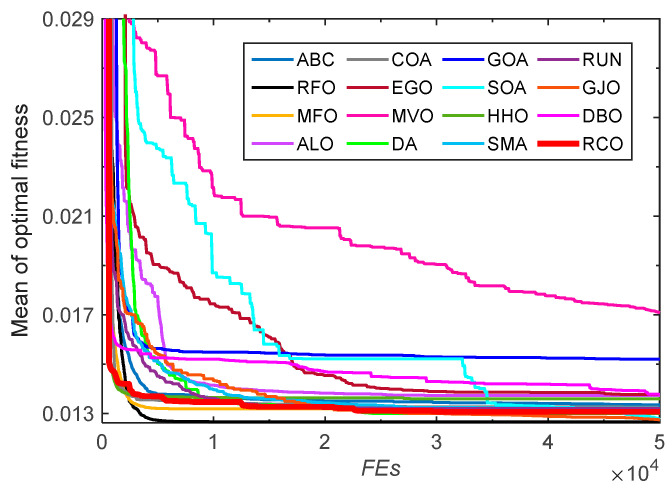
Convergence curves for spring design problem optimized by RCO and other optimizers.

**Figure A6:**
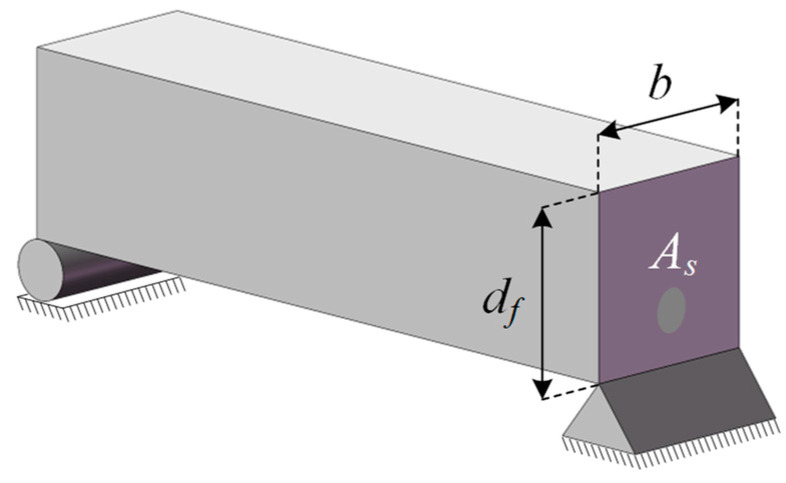
Structure diagram of reinforced concrete beam.

**Figure A7:**
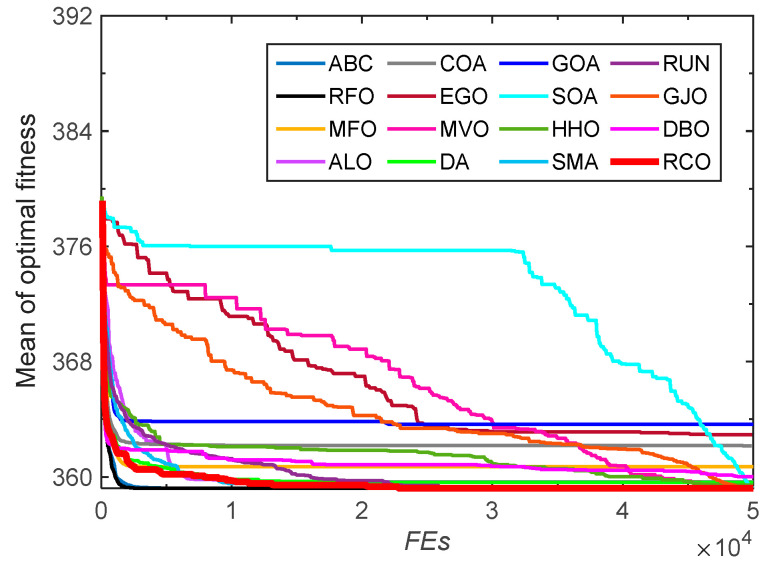
Convergence curves for reinforced concrete beam design problem optimized by RCO and other optimizers.

## Data Availability

The data presented in this study may be available on request from the corresponding author.

## Appendix A

**Table A1:** Evaluation results for CEC-2005 functions optimized by RCO and other optimizers.

	Index	RCO	DBO	GJO	RUN	SMA	HHO	COA	EGO	RFO
*F*_1_	Mean	0	5.2809 × 10^−230^	3.9820 × 10^−128^	2.2935 × 10^−243^	0	1.0477 × 10^−188^	2.7846 × 10^−300^	0	1.9440 × 10^−123^
	Std	0	2.8925 × 10^−229^	1.0427 × 10^−127^	7.3031 × 10^−243^	0	5.7385 × 10^−188^	1.5252 × 10^−299^	0	8.6274 × 10^−123^
	Min	0	0	1.9033 × 10^−132^	1.3587 × 10^−264^	0	1.1141 × 10^−218^	4.4465 × 10^−323^	0	9.2003 × 10^−147^
	Max	0	1.5843 × 10^−228^	4.1799 × 10^−127^	2.8536 × 10^−242^	0	3.1431 × 10^−187^	8.3539 × 10^−299^	0	4.7129 × 10^−122^
	Rank	1	6	8	5	1	7	4	1	9
*F*_2_	Mean	1.9434 × 10^−238^	2.3225 × 10^−127^	5.2171 × 10^−74^	1.3440 × 10^−132^	1.4401 × 10^−216^	3.6619 × 10^−102^	7.2913 × 10^−153^	6.5602 × 10^−226^	3.0528 × 10^−64^
	Std	1.0645 × 10^−237^	1.2649 × 10^−126^	1.3483 × 10^−73^	5.4701 × 10^−132^	7.8877 × 10^−216^	1.1524 × 10^−101^	2.4693 × 10^−152^	2.0974 × 10^−225^	1.0288 × 10^−63^
	Min	3.5173 × 10^−289^	1.8746 × 10^−155^	9.6739 × 10^−76^	1.1056 × 10^−144^	0	1.7402 × 10^−115^	1.7869 × 10^−161^	2.6197 × 10^−235^	3.0543 × 10^−78^
	Max	5.8303 × 10^−237^	6.9297 × 10^−126^	5.5034 × 10^−73^	2.9423 × 10^−131^	4.3203 × 10^−215^	6.0460 × 10^−101^	1.1194 × 10^−151^	9.1667 × 10^−225^	5.0084 × 10^−63^
	Rank	1	6	8	5	3	7	4	2	9
*F*_3_	Mean	0	1.2452 × 10^−108^	1.1524 × 10^−46^	2.9380 × 10^−203^	0	1.4027 × 10^−168^	1.4690 × 10^−306^	0	7.8981 × 10^−33^
	Std	0	6.8205 × 10^−108^	5.9207 × 10^−46^	1.6092 × 10^−202^	0	7.6814 × 10^−168^	5.5447 × 10^−306^	0	4.3252 × 10^−32^
	Min	0	8.7993 × 10^−296^	1.2082 × 10^−55^	5.9121 × 10^−231^	0	1.5541 × 10^−191^	5.4347 × 10^−322^	0	6.7212 × 10^−52^
	Max	0	3.7357 × 10^−107^	3.2464 × 10^−45^	8.8141 × 10^−202^	0	4.2073 × 10^−167^	2.5106 × 10^−305^	0	2.3690 × 10^−31^
	Rank	1	7	8	5	1	6	4	1	9
*F*_4_	Mean	1.3051 × 10^−226^	1.2965 × 10^−108^	1.4151 × 10^−38^	2.2911 × 10^−107^	2.4594 × 10^−209^	5.4802 × 10^−98^	3.4686 × 10^−153^	1.2982 × 10^−210^	1.2699 × 10^−30^
	Std	7.1478 × 10^−226^	7.1009 × 10^−108^	3.9775 × 10^−38^	1.2446 × 10^−106^	1.3471 × 10^−208^	1.5566 × 10^−97^	8.2139 × 10^−153^	6.0192 × 10^−210^	6.7899 × 10^−30^
	Min	1.8275 × 10^−279^	8.3784 × 10^−157^	4.6046 × 10^−41^	1.9124 × 10^−124^	0	6.2512 × 10^−110^	1.7214 × 10^−161^	1.3882 × 10^−224^	7.4726 × 10^−49^
	Max	3.9150 × 10^−225^	3.8893 × 10^−107^	2.1602 × 10^−37^	6.8188 × 10^−106^	7.3783 × 10^−208^	8.2062 × 10^−97^	2.8000 × 10^−152^	3.2877 × 10^−209^	3.7211 × 10^−29^
	Rank	1	5	8	6	3	7	4	2	9
*F*_5_	Mean	2.3255 × 10^1^	2.4222 × 10^1^	2.7537 × 10^1^	2.3002 × 10^1^	1.6940 × 10^−1^	1.4904 × 10^−3^	1.4705 × 10^−1^	2.7439 × 10^1^	1.9529 × 10^1^
	Std	1.3112 × 10^−1^	2.0856 × 10^−1^	8.1310 × 10^−1^	1.2877 × 10^0^	1.3180 × 10^−1^	2.8720 × 10^−3^	2.4898 × 10^−1^	6.2131 × 10^−1^	3.0118 × 10^0^
	Min	2.2954 × 10^1^	2.3818 × 10^1^	2.6218 × 10^1^	2.0991 × 10^1^	3.3788 × 10^−3^	1.9144 × 10^−6^	3.3091 × 10^−3^	2.6492 × 10^1^	4.1507 × 10^0^
	Max	2.3553 × 10^1^	2.4549 × 10^1^	2.8830 × 10^1^	2.5741 × 10^1^	4.7196 × 10^−1^	1.5115 × 10^−2^	1.3118 × 10^0^	2.8745 × 10^1^	2.3250 × 10^1^
	Rank	6	7	9	5	3	1	2	8	4
*F*_6_	Mean	8.2703 × 10^−8^	2.2893 × 10^−13^	2.2185 × 10^0^	1.5362 × 10^−9^	6.9817 × 10^−4^	1.1121 × 10^−5^	1.2516 × 10^−2^	4.6390 × 10^0^	3.5866 × 10^−6^
	Std	8.8799 × 10^−8^	7.0206 × 10^−13^	4.9214 × 10^−1^	6.2534 × 10^−10^	3.0287 × 10^−4^	1.5140 × 10^−5^	1.3605 × 10^−2^	4.0602 × 10^−1^	2.2533 × 10^−6^
	Min	3.5588 × 10^−9^	1.0557 × 10^−15^	1.2516 × 10^0^	6.0582 × 10^−10^	3.0562 × 10^−4^	2.2074 × 10^−8^	1.4359 × 10^−4^	3.8596 × 10^0^	8.2509 × 10^−7^
	Max	3.2621 × 10^−7^	2.9547 × 10^−12^	3.5005 × 10^0^	3.7636 × 10^−9^	1.3765 × 10^−3^	6.7073 × 10^−5^	4.4397 × 10^−2^	5.3294 × 10^0^	8.4035 × 10^−6^
	Rank	3	1	8	2	6	5	7	9	4
*F*_7_	Mean	3.8235 × 10^−5^	4.9274 × 10^−4^	1.0833 × 10^−4^	1.3639 × 10^−4^	9.4520 × 10^−5^	6.1586 × 10^−5^	3.9921 × 10^−5^	3.9288 × 10^−5^	1.9593 × 10^−3^
	Std	5.4596 × 10^−5^	4.1904 × 10^−4^	9.5409 × 10^−5^	8.4876 × 10^−5^	1.1986 × 10^−4^	8.2083 × 10^−5^	2.7439 × 10^−5^	2.7069 × 10^−5^	2.1825 × 10^−3^
	Min	5.4448 × 10^−7^	4.2673 × 10^−5^	8.5343 × 10^−6^	2.2017 × 10^−5^	4.8300 × 10^−6^	1.6027 × 10^−6^	7.4315 × 10^−7^	3.5073 × 10^−6^	1.1118 × 10^−4^
	Max	2.8157 × 10^−4^	1.5603 × 10^−3^	3.7173 × 10^−4^	3.8663 × 10^−4^	6.3406 × 10^−4^	3.6845 × 10^−4^	1.0214 × 10^−4^	9.0637 × 10^−5^	9.0398 × 10^−3^
	Rank	1	8	6	7	5	4	3	2	9
*F*_8_	Mean	−7.8058 × 10^3^	−1.0527 × 10^4^	−4.7758 × 10^3^	−8.3556 × 10^3^	−1.2569 × 10^4^	−1.2530 × 10^4^	−1.2569 × 10^4^	−7.0586 × 10^3^	−7.4069 × 10^3^
	Std	1.1148 × 10^3^	1.9844 × 10^3^	1.0318 × 10^3^	5.9000 × 10^2^	3.0944 × 10^−2^	2.1715 × 10^2^	2.8011 × 10^−2^	9.2974 × 10^2^	9.1907 × 10^2^
	Min	−1.1403 × 10^4^	−1.2562 × 10^4^	−6.5500 × 10^3^	−9.4898 × 10^3^	−1.2569 × 10^4^	−1.2569 × 10^4^	−1.2569 × 10^4^	−9.3321 × 10^3^	−9.5175 × 10^3^
	Max	−6.5455 × 10^3^	−6.8480 × 10^3^	−3.0756 × 10^3^	−6.9719 × 10^3^	−1.2569 × 10^4^	−1.1380 × 10^4^	−1.2569 × 10^4^	−5.8874 × 10^3^	−5.8374 × 10^3^
	Rank	6	4	9	5	2	3	1	8	7
*F*_9_	Mean	0	0	0	0	0	0	0	0	2.4215 × 10^0^
	Std	0	0	0	0	0	0	0	0	9.2071 × 10^0^
	Min	0	0	0	0	0	0	0	0	0
	Max	0	0	0	0	0	0	0	0	4.5768 × 10^1^
	Rank	1	1	1	1	1	1	1	1	9
*F*_10_	Mean	8.8818 × 10^−16^	8.8818 × 10^−16^	4.6777 × 10^−15^	8.8818 × 10^−16^	8.8818 × 10^−16^	8.8818 × 10^−16^	8.8818 × 10^−16^	8.8818 × 10^−16^	8.8818 × 10^−16^
	Std	0	0	9.0135 × 10^−16^	0	0	0	0	0	0
	Min	8.8818 × 10^−16^	8.8818 × 10^−16^	4.4409 × 10^−15^	8.8818 × 10^−16^	8.8818 × 10^−16^	8.8818 × 10^−16^	8.8818 × 10^−16^	8.8818 × 10^−16^	8.8818 × 10^−16^
	Max	8.8818 × 10^−16^	8.8818 × 10^−16^	7.9936 × 10^−15^	8.8818 × 10^−16^	8.8818 × 10^−16^	8.8818 × 10^−16^	8.8818 × 10^−16^	8.8818 × 10^−16^	8.8818 × 10^−16^
	Rank	1	1	9	1	1	1	1	1	1
*F*_11_	Mean	0	0	0	0	0	0	0	0	2.0490 × 10^−3^
	Std	0	0	0	0	0	0	0	0	9.1755 × 10^−3^
	Min	0	0	0	0	0	0	0	0	0
	Max	0	0	0	0	0	0	0	0	4.9149 × 10^−2^
	Rank	1	1	1	1	1	1	1	1	9
*F*_12_	Mean	2.1603 × 10^−7^	3.4556 × 10^−3^	2.3100 × 10^−1^	6.7256 × 10^−10^	3.9651 × 10^−4^	6.6063 × 10^−7^	9.6963 × 10^−5^	4.5220 × 10^−1^	2.6797 × 10^−7^
	Std	4.7274 × 10^−7^	1.8927 × 10^−2^	1.7503 × 10^−1^	1.9651 × 10^−10^	5.9390 × 10^−4^	8.9000 × 10^−7^	1.2439 × 10^−4^	7.9334 × 10^−2^	1.7045 × 10^−7^
	Min	1.2207 × 10^−8^	1.5935 × 10^−17^	9.9289 × 10^−2^	4.0781 × 10^−10^	1.2079 × 10^−6^	1.4431 × 10^−8^	1.7254 × 10^−7^	2.9122 × 10^−1^	7.2406 × 10^−8^
	Max	2.5373 × 10^−6^	1.0367 × 10^−1^	7.9104 × 10^−1^	1.2067 × 10^−9^	2.9710 × 10^−3^	4.0629 × 10^−6^	4.9112 × 10^−4^	6.3744 × 10^−1^	7.9917 × 10^−7^
	Rank	2	7	8	1	6	4	5	9	3
*F*_13_	Mean	9.7940 × 10^−1^	1.1682 × 10^−1^	1.4702 × 10^0^	5.0640 × 10^−3^	8.8519 × 10^−4^	9.0349 × 10^−6^	1.4980 × 10^−3^	7.9877 × 10^−1^	9.8447 × 10^−3^
	Std	7.1097 × 10^−1^	1.4208 × 10^−1^	2.5182 × 10^−1^	6.7515 × 10^−3^	2.0406 × 10^−3^	1.6272 × 10^−5^	2.4410 × 10^−3^	3.5452 × 10^−1^	3.0028 × 10^−2^
	Min	2.4881 × 10^−5^	4.6345 × 10^−15^	8.4171 × 10^−1^	2.2125 × 10^−9^	4.2916 × 10^−5^	5.8846 × 10^−8^	4.2391 × 10^−6^	3.1981 × 10^−1^	9.5356 × 10^−7^
	Max	2.7692 × 10^0^	6.0266 × 10^−1^	1.9765 × 10^0^	2.1024 × 10^−2^	1.1598 × 10^−2^	8.0253 × 10^−5^	1.2578 × 10^−2^	1.7298 × 10^0^	9.8924 × 10^−2^
	Rank	8	6	9	4	2	1	3	7	5
*F*_14_	Mean	9.9800 × 10^−1^	1.4887 × 10^0^	4.9781 × 10^0^	2.3436 × 10^0^	9.9800 × 10^−1^	1.0311 × 10^0^	9.9800 × 10^−1^	1.0118 × 10^0^	9.9800 × 10^−1^
	Std	2.3142 × 10^−16^	1.8652 × 10^0^	4.3820 × 10^0^	2.4550 × 10^0^	2.0785 × 10^−14^	1.8148 × 10^−1^	4.9859 × 10^−11^	5.1198 × 10^−2^	4.1233 × 10^−16^
	Min	9.9800 × 10^−1^	9.9800 × 10^−1^	9.9800 × 10^−1^	9.9800 × 10^−1^	9.9800 × 10^−1^	9.9800 × 10^−1^	9.9800 × 10^−1^	9.9800 × 10^−1^	9.9800 × 10^−1^
	Max	9.9800 × 10^−1^	1.0763 × 10^1^	1.2671 × 10^1^	1.0763 × 10^1^	9.9800 × 10^−1^	1.9920 × 10^0^	9.9800 × 10^−1^	1.2798 × 10^0^	9.9800 × 10^−1^
	Rank	1	7	9	8	3	6	4	5	2
*F*_15_	Mean	3.2153 × 10^−4^	5.8790 × 10^−4^	3.4290 × 10^−4^	7.6533 × 10^−4^	4.3310 × 10^−4^	3.5002 × 10^−4^	3.9725 × 10^−4^	3.2385 × 10^−4^	3.1037 × 10^−3^
	Std	3.5851 × 10^−5^	2.8511 × 10^−4^	1.6828 × 10^−4^	4.6567 × 10^−4^	2.0813 × 10^−4^	1.6951 × 10^−4^	1.0307 × 10^−4^	2.0846 × 10^−5^	6.8926 × 10^−3^
	Min	3.0749 × 10^−4^	3.0749 × 10^−4^	3.0749 × 10^−4^	3.0749 × 10^−4^	3.0762 × 10^−4^	3.0776 × 10^−4^	3.0995 × 10^−4^	3.0878 × 10^−4^	3.0749 × 10^−4^
	Max	4.3029 × 10^−4^	1.2239 × 10^−3^	1.2232 × 10^−3^	1.2232 × 10^−3^	1.2233 × 10^−3^	1.2437 × 10^−3^	6.7254 × 10^−4^	4.1770 × 10^−4^	2.0363 × 10^−2^
	Rank	1	7	3	8	6	4	5	2	9
*F*_16_	Mean	−1.0316 × 10^0^	−1.0316 × 10^0^	−1.0316 × 10^0^	−1.0316 × 10^0^	−1.0316 × 10^0^	−1.0316 × 10^0^	−1.0316 × 10^0^	−1.0310 × 10^0^	−1.0316 × 10^0^
	Std	5.3761 × 10^−16^	6.6486 × 10^−16^	1.8306 × 10^−8^	3.1600 × 10^−13^	1.2302 × 10^−11^	1.7724 × 10^−12^	1.4653 × 10^−4^	6.2720 × 10^−4^	6.7752 × 10^−16^
	Min	−1.0316 × 10^0^	−1.0316 × 10^0^	−1.0316 × 10^0^	−1.0316 × 10^0^	−1.0316 × 10^0^	−1.0316 × 10^0^	−1.0316 × 10^0^	−1.0316 × 10^0^	−1.0316 × 10^0^
	Max	−1.0316 × 10^0^	−1.0316 × 10^0^	−1.0316 × 10^0^	−1.0316 × 10^0^	−1.0316 × 10^0^	−1.0316 × 10^0^	−1.0308 × 10^0^	−1.0293 × 10^0^	−1.0316 × 10^0^
	Rank	1	2	7	4	6	5	8	9	3
*F*_17_	Mean	3.9789 × 10^−1^	3.9789 × 10^−1^	3.9789 × 10^−1^	3.9789 × 10^−1^	3.9789 × 10^−1^	3.9789 × 10^−1^	3.9829 × 10^−1^	3.9909 × 10^−1^	3.9789 × 10^−1^
	Std	0	0	6.5154 × 10^−6^	1.1965 × 10^−11^	2.9134 × 10^−9^	9.3218 × 10^−8^	1.3177 × 10^−3^	5.3145 × 10^−3^	0
	Min	3.9789 × 10^−1^	3.9789 × 10^−1^	3.9789 × 10^−1^	3.9789 × 10^−1^	3.9789 × 10^−1^	3.9789 × 10^−1^	3.9789 × 10^−1^	3.9789 × 10^−1^	3.9789 × 10^−1^
	Max	3.9789 × 10^−1^	3.9789 × 10^−1^	3.9791 × 10^−1^	3.9789 × 10^−1^	3.9789 × 10^−1^	3.9789 × 10^−1^	4.0455 × 10^−1^	4.2717 × 10^−1^	3.9789 × 10^−1^
	Rank	1	1	7	4	5	6	8	9	1
*F*_18_	Mean	3.0000 × 10^0^	3.0000 × 10^0^	3.0000 × 10^0^	3.0000 × 10^0^	3.0000 × 10^0^	3.0000 × 10^0^	3.0417 × 10^0^	3.0002 × 10^0^	3.0000 × 10^0^
	Std	2.0534 × 10^−15^	4.6868 × 10^−15^	7.8820 × 10^−7^	2.5021 × 10^−13^	1.3151 × 10^−11^	6.4462 × 10^−9^	1.3874 × 10^−1^	4.7576 × 10^−4^	1.2934 × 10^−15^
	Min	3.0000 × 10^0^	3.0000 × 10^0^	3.0000 × 10^0^	3.0000 × 10^0^	3.0000 × 10^0^	3.0000 × 10^0^	3.0000 × 10^0^	3.0000 × 10^0^	3.0000 × 10^0^
	Max	3.0000 × 10^0^	3.0000 × 10^0^	3.0000 × 10^0^	3.0000 × 10^0^	3.0000 × 10^0^	3.0000 × 10^0^	3.7212 × 10^0^	3.0021 × 10^0^	3.0000 × 10^0^
	Rank	2	3	7	4	5	6	9	8	1
*F*_19_	Mean	−3.8628 × 10^0^	−3.8615 × 10^0^	−3.8612 × 10^0^	−3.8628 × 10^0^	−3.8628 × 10^0^	−3.8624 × 10^0^	−3.8430 × 10^0^	−3.8591 × 10^0^	−3.8628 × 10^0^
	Std	2.2494 × 10^−15^	2.9649 × 10^−3^	3.1976 × 10^−3^	3.1487 × 10^−10^	1.0144 × 10^−8^	6.4592 × 10^−4^	2.5786 × 10^−2^	2.9782 × 10^−3^	2.7101 × 10^−15^
	Min	−3.8628 × 10^0^	−3.8628 × 10^0^	−3.8628 × 10^0^	−3.8628 × 10^0^	−3.8628 × 10^0^	−3.8628 × 10^0^	−3.8628 × 10^0^	−3.8625 × 10^0^	−3.8628 × 10^0^
	Max	−3.8628 × 10^0^	−3.8549 × 10^0^	−3.8549 × 10^0^	−3.8628 × 10^0^	−3.8628 × 10^0^	−3.8602 × 10^0^	−3.7757 × 10^0^	−3.8544 × 10^0^	−3.8628 × 10^0^
	Rank	1	6	8	3	4	5	9	7	2
*F*_20_	Mean	−3.2863 × 10^0^	−3.2362 × 10^0^	−3.1541 × 10^0^	−3.2507 × 10^0^	−3.2189 × 10^0^	−3.2101 × 10^0^	−2.8638 × 10^0^	−3.2469 × 10^0^	−3.2508 × 10^0^
	Std	5.5415 × 10^−2^	8.4116 × 10^−2^	1.3116 × 10^−1^	5.9241 × 10^−2^	4.1112 × 10^−2^	6.5791 × 10^−2^	2.6506 × 10^−1^	8.9701 × 10^−2^	5.9397 × 10^−2^
	Min	−3.3220 × 10^0^	−3.3220 × 10^0^	−3.3220 × 10^0^	−3.3220 × 10^0^	−3.3220 × 10^0^	−3.3172 × 10^0^	−3.2038 × 10^0^	−3.3133 × 10^0^	−3.3224 × 10^0^
	Max	−3.2031 × 10^0^	−3.0839 × 10^0^	−2.6381 × 10^0^	−3.2031 × 10^0^	−3.2027 × 10^0^	−3.0883 × 10^0^	−2.1416 × 10^0^	−3.0449 × 10^0^	−3.2032 × 10^0^
	Rank	1	5	8	3	6	7	9	4	2
*F*_21_	Mean	−1.0153 × 10^1^	−7.6162 × 10^0^	−9.0867 × 10^0^	−1.0153 × 10^1^	−1.0153 × 10^1^	−5.3899 × 10^0^	−1.0153 × 10^1^	−5.3010 × 10^0^	−8.0521 × 10^0^
	Std	6.1269 × 10^−15^	2.5802 × 10^0^	2.1780 × 10^0^	5.1533 × 10^−10^	6.3068 × 10^−5^	1.2815 × 10^0^	1.1031 × 10^−4^	1.0508 × 10^0^	2.8888 × 10^0^
	Min	−1.0153 × 10^1^	−1.0153 × 10^1^	−1.0153 × 10^1^	−1.0153 × 10^1^	−1.0153 × 10^1^	−1.0135 × 10^1^	−1.0153 × 10^1^	−9.8387 × 10^0^	−1.0153 × 10^1^
	Max	−1.0153 × 10^1^	−5.0551 × 10^0^	−3.5916 × 10^0^	−1.0153 × 10^1^	−1.0153 × 10^1^	−5.0476 × 10^0^	−1.0153 × 10^1^	−4.9663 × 10^0^	−2.6305 × 10^0^
	Rank	1	7	5	2	3	8	4	9	6
*F*_22_	Mean	−1.0403 × 10^1^	−7.6775 × 10^0^	−9.9734 × 10^0^	−1.0403 × 10^1^	−1.0403 × 10^1^	−5.2615 × 10^0^	−1.0403 × 10^1^	−5.2316 × 10^0^	−8.9356 × 10^0^
	Std	2.4240 × 10^−15^	2.8037 × 10^0^	1.6514 × 10^0^	4.0533 × 10^−10^	4.1346 × 10^−5^	9.6677 × 10^−1^	9.3489 × 10^−5^	9.3835 × 10^−1^	2.7659 × 10^0^
	Min	−1.0403 × 10^1^	−1.0403 × 10^1^	−1.0403 × 10^1^	−1.0403 × 10^1^	−1.0403 × 10^1^	−1.0380 × 10^1^	−1.0403 × 10^1^	−1.0199 × 10^1^	−1.0403 × 10^1^
	Max	−1.0403 × 10^1^	−2.7659 × 10^0^	−2.8676 × 10^0^	−1.0403 × 10^1^	−1.0403 × 10^1^	−5.0687 × 10^0^	−1.0402 × 10^1^	−5.0158 × 10^0^	−2.7519 × 10^0^
	Rank	1	7	5	2	3	8	4	9	6
*F*_23_	Mean	−1.0536 × 10^1^	−8.9233 × 10^0^	−9.3781 × 10^0^	−1.0536 × 10^1^	−1.0536 × 10^1^	−5.3034 × 10^0^	−1.0536 × 10^1^	−5.2654 × 10^0^	−8.2315 × 10^0^
	Std	4.6181 × 10^−15^	2.5060 × 10^0^	2.6826 × 10^0^	2.9870 × 10^−10^	3.5879 × 10^−5^	9.7000 × 10^−1^	7.3804 × 10^−5^	8.5743 × 10^−1^	3.3811 × 10^0^
	Min	−1.0536 × 10^1^	−1.0536 × 10^1^	−1.0536 × 10^1^	−1.0536 × 10^1^	−1.0536 × 10^1^	−1.0439 × 10^1^	−1.0536 × 10^1^	−9.8046 × 10^0^	−1.0536 × 10^1^
	Max	−1.0536 × 10^1^	−5.1285 × 10^0^	−2.4216 × 10^0^	−1.0536 × 10^1^	−1.0536 × 10^1^	−5.1086 × 10^0^	−1.0536 × 10^1^	−5.0699 × 10^0^	−2.4217 × 10^0^
	Rank	1	6	5	2	3	8	4	9	7
Mean rank		2.5000	5.3261	7.0870	4.2826	3.9783	5.2826	4.9783	5.8913	5.6739
Total rank		1	6	9	3	2	5	4	8	7

**Table A2:** Average running time (s) for CEC-2005 functions optimized by RCO and other optimizers.

	RCO	DBO	GJO	RUN	SMA	HHO	COA	EGO	RFO
*F*_1_	0.2193	0.2014	0.2639	1.1369	0.8407	0.2436	0.1278	0.1708	0.3725
*F*_2_	0.2248	0.2036	0.2619	1.0974	0.7864	0.2292	0.1346	0.1895	0.3849
*F*_3_	0.5951	0.5736	0.6648	1.7278	1.1698	1.1522	0.6970	0.5200	0.7107
*F*_4_	0.2137	0.1979	0.2523	1.0689	0.7894	0.2804	0.1250	0.1648	0.3703
*F*_5_	0.2560	0.2415	0.2989	1.1596	0.8604	0.4365	0.1901	0.2065	0.4073
*F*_6_	0.2137	0.2070	0.2526	1.0572	0.8014	0.3303	0.1240	0.1776	0.3688
*F*_7_	0.3947	0.3755	0.4437	1.3862	0.9758	0.6812	0.4043	0.3431	0.5414
*F*_8_	0.2598	0.2659	0.3086	1.1745	0.8498	0.4571	0.1896	0.2086	0.3960
*F*_9_	0.2386	0.2120	0.2674	1.1250	0.8085	0.3817	0.1579	0.1823	0.3984
*F*_10_	0.2437	0.2220	0.2707	1.1269	0.8099	0.3928	0.1690	0.1983	0.4053
*F*_11_	0.2707	0.2672	0.3163	1.1852	0.8394	0.4590	0.2190	0.2228	0.4342
*F*_12_	0.7002	0.6956	0.8083	1.9595	1.3313	1.5442	0.9279	0.6701	0.8538
*F*_13_	0.7121	0.7053	0.8029	1.9861	1.2753	1.5663	0.9374	0.6804	1.1196
*F*_14_	1.0432	1.1472	1.0631	2.6387	1.3272	2.6602	1.5988	0.9993	1.2211
*F*_15_	0.1188	0.1918	0.1538	0.9414	0.4131	0.2722	0.1372	0.0949	0.3190
*F*_16_	0.1046	0.1737	0.1422	0.9128	0.3749	0.2630	0.1259	0.0855	0.3233
*F*_17_	0.0901	0.1721	0.1323	0.8944	0.3612	0.2314	0.1006	0.0906	0.3102
*F*_18_	0.0875	0.1585	0.1239	0.9066	0.3632	0.2248	0.0997	0.0705	0.3057
*F*_19_	0.1249	0.2003	0.1657	0.9690	0.4027	0.3106	0.1541	0.1048	0.3318
*F*_20_	0.1352	0.2021	0.1857	0.9752	0.4621	0.3217	0.1562	0.1138	0.3544
*F*_21_	0.1321	0.2016	0.1694	1.0126	0.4204	0.3049	0.1503	0.1067	0.3338
*F*_22_	0.1360	0.2137	0.1808	1.0024	0.4532	0.3254	0.1689	0.1192	0.3528
*F*_23_	0.1473	0.2197	0.1942	1.0146	0.4502	0.3686	0.1908	0.1292	0.3616

**Table A3:** Evaluation results for CEC-2022 test functions optimized by RCO and other optimizers.

	Index	RCO	DBO	GJO	RUN	SMA	HHO	COA	EGO	RFO
*F*_24_	Mean	3.0000 × 10^2^	3.0000 × 10^2^	2.5036 × 10^3^	3.0000 × 10^2^	3.0000 × 10^2^	3.0066 × 10^2^	4.6737 × 10^2^	6.8079 × 10^2^	3.1808 × 10^2^
	Std	9.5520 × 10^−7^	6.5870 × 10^−3^	2.1924 × 10^3^	1.1582 × 10^−4^	1.8839 × 10^−4^	2.6587 × 10^−1^	6.7693 × 10^1^	1.0234 × 10^2^	4.9090 × 10^1^
	Min	3.0000 × 10^2^	3.0000 × 10^2^	4.3993 × 10^2^	3.0000 × 10^2^	3.0000 × 10^2^	3.0025 × 10^2^	3.2378 × 10^2^	4.8892 × 10^2^	3.0000 × 10^2^
	Max	3.0000 × 10^2^	3.0004 × 10^2^	8.5756 × 10^3^	3.0000 × 10^2^	3.0000 × 10^2^	3.0144 × 10^2^	6.5187 × 10^2^	8.7563 × 10^2^	5.6092 × 10^2^
	Rank	1	4	9	2	3	5	7	8	6
*F*_2__5_	Mean	4.0845 × 10^2^	4.2628 × 10^2^	4.4023 × 10^2^	4.0961 × 10^2^	4.0963 × 10^2^	4.1502 × 10^2^	4.3332 × 10^2^	4.2556 × 10^2^	4.0938 × 10^2^
	Std	1.3978 × 10^1^	3.1933 × 10^1^	2.8100 × 10^1^	1.7157 × 10^1^	1.2213 × 10^1^	2.1922 × 10^1^	3.0829 × 10^1^	2.2737 × 10^1^	4.4093 × 10^0^
	Min	4.0000 × 10^2^	4.0012 × 10^2^	4.0644 × 10^2^	4.0000 × 10^2^	4.0564 × 10^2^	4.0004 × 10^2^	4.0020 × 10^2^	4.0062 × 10^2^	4.0014 × 10^2^
	Max	4.6894 × 10^2^	4.9270 × 10^2^	5.2571 × 10^2^	4.7078 × 10^2^	4.7393 × 10^2^	4.7104 × 10^2^	4.8553 × 10^2^	4.7090 × 10^2^	4.1946 × 10^2^
	Rank	1	7	9	3	4	5	8	6	2
*F*_2__6_	Mean	6.1579 × 10^2^	6.2004 × 10^2^	6.3574 × 10^2^	6.1663 × 10^2^	6.0007 × 10^2^	6.2819 × 10^2^	6.1631 × 10^2^	6.1688 × 10^2^	6.0528 × 10^2^
	Std	9.8711 × 10^0^	9.7215 × 10^0^	7.5118 × 10^0^	8.6491 × 10^0^	1.4000 × 10^−1^	1.2081 × 10^1^	7.7566 × 10^0^	4.0105 × 10^0^	3.2735 × 10^0^
	Min	6.0163 × 10^2^	6.0342 × 10^2^	6.2369 × 10^2^	6.0322 × 10^2^	6.0002 × 10^2^	6.0546 × 10^2^	6.0192 × 10^2^	6.1177 × 10^2^	6.0035 × 10^2^
	Max	6.3772 × 10^2^	6.3747 × 10^2^	6.5486 × 10^2^	6.3448 × 10^2^	6.0081 × 10^2^	6.5478 × 10^2^	6.3764 × 10^2^	6.2980 × 10^2^	6.1104 × 10^2^
	Rank	3	7	9	5	1	8	4	6	2
*F*_2__7_	Mean	8.2262 × 10^2^	8.2519 × 10^2^	8.2548 × 10^2^	8.2315 × 10^2^	8.2322 × 10^2^	8.2463 × 10^2^	8.4184 × 10^2^	8.2900 × 10^2^	8.2810 × 10^2^
	Std	9.1560 × 10^0^	9.2451 × 10^0^	7.7796 × 10^0^	6.3624 × 10^0^	1.0028 × 10^1^	6.6644 × 10^0^	1.5499 × 10^1^	1.1551 × 10^1^	1.2351 × 10^1^
	Min	8.0796 × 10^2^	8.0791 × 10^2^	8.1225 × 10^2^	8.1094 × 10^2^	8.0697 × 10^2^	8.1300 × 10^2^	8.1892 × 10^2^	8.1094 × 10^2^	8.0895 × 10^2^
	Max	8.3582 × 10^2^	8.3811 × 10^2^	8.4503 × 10^2^	8.3383 × 10^2^	8.4378 × 10^2^	8.4099 × 10^2^	8.7466 × 10^2^	8.5373 × 10^2^	8.5115 × 10^2^
	Rank	1	5	6	2	3	4	9	8	7
*F*_2__8_	Mean	9.9915 × 10^2^	1.0588 × 10^3^	1.1603 × 10^3^	1.0233 × 10^3^	9.0018 × 10^2^	1.3088 × 10^3^	1.0078 × 10^3^	1.0060 × 10^3^	9.2283 × 10^2^
	Std	1.3394 × 10^2^	1.2952 × 10^2^	1.5648 × 10^2^	7.4703 × 10^1^	2.8177 × 10^−1^	1.7534 × 10^2^	5.7384 × 10^1^	2.3982 × 10^2^	4.2240 × 10^1^
	Min	9.0000 × 10^2^	9.0018 × 10^2^	9.8482 × 10^2^	9.4077 × 10^2^	9.0000 × 10^2^	1.0097 × 10^3^	9.0539 × 10^2^	9.0000 × 10^2^	9.0010 × 10^2^
	Max	1.3612 × 10^3^	1.4457 × 10^3^	1.5323 × 10^3^	1.2464 × 10^3^	9.0091 × 10^2^	1.6358 × 10^3^	1.1268 × 10^3^	1.9679 × 10^3^	1.0671 × 10^3^
	Rank	3	7	8	6	1	9	5	4	2
*F*_2__9_	Mean	3.3257 × 10^3^	4.8438 × 10^3^	7.1368 × 10^3^	3.3307 × 10^3^	5.9396 × 10^3^	3.7954 × 10^3^	3.7087 × 10^3^	4.9859 × 10^3^	5.0525 × 10^3^
	Std	1.4167 × 10^3^	2.1609 × 10^3^	1.8322 × 10^3^	1.3872 × 10^3^	2.0529 × 10^3^	2.3647 × 10^3^	1.5260 × 10^3^	2.2015 × 10^3^	2.2488 × 10^3^
	Min	1.8825 × 10^3^	1.9222 × 10^3^	2.6176 × 10^3^	1.8847 × 10^3^	1.9655 × 10^3^	1.9369 × 10^3^	1.9336 × 10^3^	1.8568 × 10^3^	1.8341 × 10^3^
	Max	6.4416 × 10^3^	8.2446 × 10^3^	8.9817 × 10^3^	7.2376 × 10^3^	8.1397 × 10^3^	8.2625 × 10^3^	8.0003 × 10^3^	8.1304 × 10^3^	8.2965 × 10^3^
	Rank	1	5	9	2	8	4	3	6	7
*F*_30_	Mean	2.0399 × 10^3^	2.0463 × 10^3^	2.0444 × 10^3^	2.0392 × 10^3^	2.0188 × 10^3^	2.0506 × 10^3^	2.0410 × 10^3^	2.0511 × 10^3^	2.0170 × 10^3^
	Std	2.0802 × 10^1^	1.9596 × 10^1^	1.9758 × 10^1^	1.2284 × 10^1^	5.9280 × 10^0^	2.5132 × 10^1^	1.4021 × 10^1^	8.0569 × 10^0^	9.2755 × 10^0^
	Min	2.0139 × 10^3^	2.0230 × 10^3^	2.0140 × 10^3^	2.0103 × 10^3^	2.0000 × 10^3^	2.0247 × 10^3^	2.0200 × 10^3^	2.0362 × 10^3^	2.0010 × 10^3^
	Max	2.0923 × 10^3^	2.0995 × 10^3^	2.1126 × 10^3^	2.0653 × 10^3^	2.0226 × 10^3^	2.1135 × 10^3^	2.0732 × 10^3^	2.0693 × 10^3^	2.0300 × 10^3^
	Rank	4	7	6	3	2	8	5	9	1
*F*_31_	Mean	2.2273 × 10^3^	2.2276 × 10^3^	2.2264 × 10^3^	2.2226 × 10^3^	2.2207 × 10^3^	2.2295 × 10^3^	2.2312 × 10^3^	2.2266 × 10^3^	2.2307 × 10^3^
	Std	5.2714 × 10^0^	6.8296 × 10^0^	3.5636 × 10^0^	3.8180 × 10^0^	4.9956 × 10^−1^	1.2234 × 10^1^	2.9311 × 10^0^	5.6304 × 10^0^	6.8252 × 10^0^
	Min	2.2058 × 10^3^	2.2124 × 10^3^	2.2206 × 10^3^	2.2041 × 10^3^	2.2200 × 10^3^	2.2075 × 10^3^	2.2227 × 10^3^	2.2126 × 10^3^	2.2121 × 10^3^
	Max	2.2340 × 10^3^	2.2479 × 10^3^	2.2332 × 10^3^	2.2254 × 10^3^	2.2216 × 10^3^	2.2638 × 10^3^	2.2389 × 10^3^	2.2319 × 10^3^	2.2505 × 10^3^
	Rank	5	6	3	2	1	7	9	4	8
*F*_32_	Mean	2.5293 × 10^3^	2.5308 × 10^3^	2.5826 × 10^3^	2.5293 × 10^3^	2.5293 × 10^3^	2.5346 × 10^3^	2.5595 × 10^3^	2.5542 × 10^3^	2.5360 × 10^3^
	Std	2.6704 × 10^−13^	4.6347 × 10^0^	3.3278 × 10^1^	2.5820 × 10^−4^	9.2424 × 10^−5^	2.6781 × 10^1^	1.5811 × 10^1^	1.3486 × 10^1^	1.7542 × 10^1^
	Min	2.5293 × 10^3^	2.5293 × 10^3^	2.5306 × 10^3^	2.5293 × 10^3^	2.5293 × 10^3^	2.5293 × 10^3^	2.5385 × 10^3^	2.5352 × 10^3^	2.5293 × 10^3^
	Max	2.5293 × 10^3^	2.5483 × 10^3^	2.6734 × 10^3^	2.5293 × 10^3^	2.5293 × 10^3^	2.6762 × 10^3^	2.5980 × 10^3^	2.5905 × 10^3^	2.6183 × 10^3^
	Rank	1	4	9	3	2	5	8	7	6
*F*_33_	Mean	2.5006 × 10^3^	2.5179 × 10^3^	2.5667 × 10^3^	2.5469 × 10^3^	2.5079 × 10^3^	2.5444 × 10^3^	2.5113 × 10^3^	2.5062 × 10^3^	2.5434 × 10^3^
	Std	1.6487 × 10^−1^	4.4319 × 10^1^	6.2210 × 10^1^	5.7549 × 10^1^	2.8585 × 10^1^	6.3047 × 10^1^	3.5215 × 10^1^	2.5066 × 10^1^	5.7762 × 10^1^
	Min	2.5003 × 10^3^	2.5003 × 10^3^	2.5003 × 10^3^	2.5004 × 10^3^	2.5003 × 10^3^	2.5004 × 10^3^	2.5009 × 10^3^	2.5008 × 10^3^	2.5001 × 10^3^
	Max	2.5010 × 10^3^	2.6313 × 10^3^	2.6347 × 10^3^	2.6246 × 10^3^	2.6138 × 10^3^	2.6482 × 10^3^	2.6511 × 10^3^	2.6388 × 10^3^	2.6311 × 10^3^
	Rank	1	5	9	8	3	7	4	2	6
*F*_34_	Mean	2.7510 × 10^3^	2.7715 × 10^3^	2.9569 × 10^3^	2.7450 × 10^3^	2.7549 × 10^3^	2.8005 × 10^3^	2.8759 × 10^3^	2.8192 × 10^3^	2.9846 × 10^3^
	Std	1.5730 × 10^2^	2.5274 × 10^2^	2.2904 × 10^2^	1.4993 × 10^2^	1.9730 × 10^2^	1.5457 × 10^2^	1.6812 × 10^2^	1.0886 × 10^2^	1.8435 × 10^2^
	Min	2.6000 × 10^3^	2.6000 × 10^3^	2.6040 × 10^3^	2.6000 × 10^3^	2.6000 × 10^3^	2.6033 × 10^3^	2.7700 × 10^3^	2.7614 × 10^3^	2.6038 × 10^3^
	Max	3.0000 × 10^3^	3.9036 × 10^3^	3.4254 × 10^3^	2.9002 × 10^3^	3.2127 × 10^3^	3.2133 × 10^3^	3.3418 × 10^3^	3.2675 × 10^3^	3.4250 × 10^3^
	Rank	2	4	8	1	3	5	7	6	9
*F*_35_	Mean	2.8656 × 10^3^	2.8678 × 10^3^	2.8701 × 10^3^	2.8637 × 10^3^	2.8619 × 10^3^	2.9049 × 10^3^	2.9244 × 10^3^	2.8933 × 10^3^	2.8737 × 10^3^
	Std	1.6380 × 10^0^	7.3106 × 10^0^	1.2778 × 10^1^	1.7089 × 10^0^	1.4949 × 10^0^	4.4703 × 10^1^	2.5242 × 10^1^	1.0017 × 10^1^	1.0000 × 10^1^
	Min	2.8626 × 10^3^	2.8597 × 10^3^	2.8586 × 10^3^	2.8586 × 10^3^	2.8586 × 10^3^	2.8649 × 10^3^	2.8810 × 10^3^	2.8721 × 10^3^	2.8635 × 10^3^
	Max	2.8682 × 10^3^	2.8994 × 10^3^	2.9158 × 10^3^	2.8665 × 10^3^	2.8639 × 10^3^	3.0521 × 10^3^	2.9836 × 10^3^	2.9000 × 10^3^	2.9012 × 10^3^
	Rank	3	4	5	2	1	8	9	7	6
Mean rank		2.1667	5.4167	7.5000	3.2500	2.6667	6.2500	6.5000	6.0833	5.1667
Total rank		1	5	9	3	2	7	8	6	4

## Appendix B

### Appendix B.1. Himmelblau’s Nonlinear Problem

Himmelblau’s nonlinear problem is a typical optimization problem with constraints, and thus we attempt to solve it using the proposed RCO algorithm. This problem contains five decision variables, and its goal is to minimize the objective function subject to six inequality constraints. It can be described as follows:

Consider variable $X=\left[x_{1},x_{2},x_{3},x_{4},x_{5}\right]$;

minimize $F(X)=5.3578547x_{3}^{2}+0.8356891x_{1}x_{5}+37.293239x_{1}-40792.141$;

subject to

$$g_{1}(X)=-85.334407-0.0056858x_{2}x_{5}-0.0006262x_{1}x_{4}+0.0022053x_{3}x_{5}\leq 0,$$

$$g_{2}(X)=85.334407+0.0056858x_{2}x_{5}+0.0006262x_{1}x_{4}-0.0022053x_{3}x_{5}-92\leq 0,$$

$$g_{3}(X)=-80.51249-0.0071317x_{2}x_{5}-0.0029955x_{1}x_{2}-0.0021813x_{3}^{2}+90\leq 0,$$

$$g_{4}(X)=80.51249+0.0071317x_{2}x_{5}+0.0029955x_{1}x_{2}+0.0021813x_{3}^{2}-110\leq 0,$$

$$g_{5}(X)=-9.300961-0.0047026x_{3}x_{5}-0.0012547x_{1}x_{3}-0.0019085x_{3}x_{4}+20\leq 0,$$

$$g_{6}(X)=9.300961+0.0047026x_{3}x_{5}+0.0012547x_{1}x_{3}+0.0019085x_{3}x_{4}-25\leq 0;$$

variable range: $78\leq x_{1}\leq 102$, $33\leq x_{2}\leq 45$, $27\leq x_{3},x_{4},x_{5}\leq 45$.

Table A4 shows the evaluation results of RCO and other algorithms for optimizing this problem. It can be found that RCO, DBO, MFO, and RFO give the minimum value of the objective function in 30 repeated attempts. The optimal value is *F*(*X*) = −30665.53867178, and the corresponding optimal solution is *X* = (78, 33, 29.995256025680, 45, 36.775812905789). Further comparisons reveal that RCO outperforms DBO, MFO, and RFO in terms of mean and standard deviation indicators. Therefore, RCO ranks first in responding to this problem. In addition, RCO has a faster convergence rate compared to most of the algorithms mentioned above, as shown in Figure A1.

**Table A4:** Evaluation results for Himmelblau’s nonlinear problem optimized by RCO and other optimizers.

Optimizer	Mean	Std	Best	Worst
RCO	−30,665.32349854	7.1895 × 10^−1^	−30,665.53867178	−30,661.79892780
DBO	−30,635.71460849	1.6335 × 10^2^	−30,665.53867178	−29,770.81677325
GJO	−30,657.37893505	4.1842 × 10^0^	−30,663.55315586	−30,646.38130073
RUN	−30,660.13539140	1.9969 × 10^1^	−30,665.53586489	−30,561.69882047
SMA	−30,665.53734918	1.5540 × 10^−3^	−30,665.53865029	−30,665.53262256
HHO	−30,532.30441778	1.5263 × 10^2^	−30,663.48977472	−30,201.43851773
SOA	−30,641.70338442	1.7341 × 10^1^	−30,657.74185848	−30,566.54192773
GOA	−30,496.99193113	2.2198 × 10^2^	−30,665.43888010	−29,837.56764245
DA	−30,625.26683639	9.1543 × 10^1^	−30,665.53866631	−30,342.54049981
MVO	−30,575.23585370	7.9803 × 10^1^	−30,662.44936380	−30,386.62854427
EGO	−30,354.73643722	1.5849 × 10^2^	−30,634.70568304	−30,085.99212698
COA	−30,221.79295008	2.7718 × 10^2^	−30,660.04569407	−29,657.05292693
ALO	−30,624.67887473	1.0054 × 10^2^	−30,665.53858867	−30,218.93339012
MFO	−30,665.31055628	1.2452 × 10^0^	−30,665.53867178	−30,658.71750920
RFO	−30,658.81428956	1.8304 × 10^1^	−30,665.53867178	−30,572.05049500
ABC	−30,607.67379908	1.1696 × 10^1^	−30,630.43422662	−30,584.93003770

### Appendix B.2. I-Beam Design Problem

I-beams are widely used in the construction industry. The I-beam design problem is to minimize the vertical deflection of the beam while meeting the stress and cross-sectional area constraints under the required loads. As shown in Figure A2, it contains four decision variables: the width of beam *b*, the height of beam *h*, the thickness of waist *t_w_*, and the thickness of foot *t_f_*. The complete description of this problem is as follows:

Consider variable $X=\left[x_{1},x_{2},x_{3},x_{4}\right]=\left[b,h,t_{w},t_{f}\right]$;

minimize $F(X)=\frac{5000}{x_{3}{\left(x_{2}-2x_{4}\right)}^{3}/12+\left(x_{1}x_{4}^{3}\right)/6+2x_{1}x_{4}{\left(\left(x_{2}-x_{4}\right)/2\right)}^{2}}$;

subject to

$$g_{1}(X)=2x_{1}x_{4}+x_{3}\left(x_{2}-2x_{4}\right)-300\leq 0,$$

$$g_{2}(X)=\frac{180000x_{2}}{x_{3}{\left(x_{2}-2x_{4}\right)}^{3}+2x_{1}x_{4}\left(4x_{4}^{2}+3x_{2}\left(x_{2}-2x_{4}\right)\right)}+\frac{15000x_{1}}{x_{3}^{3}\left(x_{2}-2x_{4}\right)+2x_{1}^{3}x_{4}}-16\leq 0;$$

variable range: $10\leq x_{1}\leq 50$, $10\leq x_{2}\leq 80$, $0.9\leq x_{3},x_{4}\leq 5$.

According to the evaluation results shown in Table A5, it is found that RCO, DBO, HHO, GOA, DA, MFO, and RFO can successfully solve this problem. The optimal solution obtained is *X* = (50, 80, 0.9, 2.321792260692), which achieves the minimum vertical deflection *F*(*X*) = 0.013074118905. Furthermore, the proposed RCO returns better mean and standard deviation values compared to the other six algorithms, which demonstrates its advantages and stability. Figure A3 shows that RCO not only has high accuracy but also has a fast convergence rate in solving the I-beam problem.

**Table A5:** Evaluation results for I-beam design problem optimized by RCO and other optimizers.

Optimizer	Mean	Std	Best	Worst
RCO	0.013074120217	7.0324 × 10^−9^	0.013074118905	0.013074157447
DBO	0.013129724700	2.1161 × 10^−4^	0.013074118905	0.013908205841
GJO	0.013074931995	6.2377 × 10^−7^	0.013074156740	0.013076087308
RUN	0.013074121447	2.4426 × 10^−9^	0.013074118987	0.013074128823
SMA	0.013074122152	1.3191 × 10^−8^	0.013074118913	0.013074191503
HHO	0.013075343023	5.1039 × 10^−6^	0.013074118905	0.013102198502
SOA	0.013076269212	2.0988 × 10^−6^	0.013074133499	0.013081247447
GOA	0.013074159564	2.1984 × 10^−7^	0.013074118905	0.013075323503
DA	0.013114850040	1.6511 × 10^−4^	0.013074118905	0.013908205863
MVO	0.013075030628	9.5513 × 10^−7^	0.013074134449	0.013078209725
EGO	0.013186062373	1.8456 × 10^−4^	0.013075003778	0.013818841015
COA	0.013156084492	2.3038 × 10^−4^	0.013074288457	0.013915045976
ALO	0.013080104310	1.7143 × 10^−5^	0.013074118913	0.013151854904
MFO	0.013083109110	4.8065 × 10^−5^	0.013074118905	0.013337561384
RFO	0.013074170655	8.5477 × 10^−8^	0.013074118905	0.013075136549
ABC	0.013247028789	3.3982 × 10^−5^	0.013139820756	0.013264707253

### Appendix B.3. Tension/Compression Spring Design Problem

The spring design problem has been optimized by many researchers using their methods and is therefore also optimized here. The schematic diagram of a tension/compression spring is shown in Figure A4. The requirement of this problem is to find the solution that can minimize the weight of the spring on the premise of satisfying four inequality constraints. This problem includes three variables: the wire diameter *d*, the mean coil diameter *D*, and the number of active coils *N*. Its design problem is modeled as follows:

Consider variable $X=\left[x_{1},x_{2},x_{3}\right]=\left[d,D,N\right]$;

minimize $F(X)=x_{1}^{2}x_{2}\left(x_{3}+2\right)$;

subject to

$$g_{1}(X)=1-\frac{x_{2}^{3}x_{3}}{71785x_{1}^{4}}\leq 0,$$

$$g_{2}(X)=\frac{4x_{2}^{2}-x_{1}x_{2}}{12566\left(x_{1}^{3}x_{2}-x_{1}^{4}\right)}+\frac{1}{5108x_{1}^{2}}-1\leq 0,$$

$$g_{3}(X)=1-\frac{140.45x_{1}}{x_{2}^{2}x_{3}}\leq 0,$$

$$g_{4}(X)=\frac{x_{1}+x_{2}}{1.5}-1\leq 0;$$

variable range: $0.05\leq x_{1}\leq 2$, $0.25\leq x_{2}\leq 1.3$, $2\leq x_{3}\leq 15$.

The evaluation results of all optimizers on the spring design problem are presented in Table A6. From the indicator of the best value, it is found that RCO, DBO, and RFO give very close results, which are better than the other algorithms. In more detail, the best solution obtained by RCO is slightly worse than RFO. This solution is *X* = (0.051696624950, 0.356899733826, 11.278303978922), achieving a minimum weight of *F*(*X*) = 0.012665233831. However, the RCO is mediocre in terms of mean value performance. The convergence curves of RCO and other optimizers are shown in Figure A5. Overall, the best solution found by RCO in multiple runs and the convergence behavior are satisfactory.

**Table A6:** Evaluation results for spring design problem optimized by RCO and other optimizers.

Optimizer	Mean	Std	Best	Worst
RCO	0.013053427666	7.8521 × 10^−4^	0.012665233831	0.015754390208
DBO	0.013772440263	1.8547 × 10^−3^	0.012665304859	0.017773158078
GJO	0.012736806942	4.4089 × 10^−5^	0.012684425803	0.012958545486
RUN	0.013197665660	1.0827 × 10^−3^	0.012666334447	0.017773164310
SMA	0.013213244427	7.3444 × 10^−4^	0.012669726461	0.015376704673
HHO	0.013597048937	9.5390 × 10^−4^	0.012666237221	0.017774812796
SOA	0.012752131110	2.2074 × 10^−5^	0.012704757407	0.012811498589
GOA	0.015198086272	1.9339 × 10^−3^	0.012668078008	0.017867196026
DA	0.012991820530	4.2921 × 10^−4^	0.012689820508	0.014901052692
MVO	0.017090902859	1.6960 × 10^−3^	0.012761974498	0.018383176280
EGO	0.013772491910	9.6876 × 10^−4^	0.012727047892	0.016798046250
COA	0.013258265593	8.7897 × 10^−4^	0.012685526191	0.017299772980
ALO	0.013724936736	1.5357 × 10^−3^	0.012672418461	0.017773186025
MFO	0.013188626844	1.0021 × 10^−3^	0.012667085044	0.017772992685
RFO	0.012665232789	5.6062 × 10^−13^	0.012665232788	0.012665232791
ABC	0.013349426786	2.4523 × 10^−4^	0.012865635902	0.013783878098

### Appendix B.4. Reinforced Concrete Beam Design Problem

The decision variables of several engineering application problems mentioned above are continuous. Unlike them, discrete variables appear in the reinforced concrete beam design problem. The problem aims to save the total cost of reinforcement and concrete as much as possible. Figure A6 shows the schematic diagram of a reinforced concrete beam, which includes two discrete variables (the area of reinforcement *A_s_* and the depth of beam *d_f_*) and one continuous variable (the width of beam *b*). The optimization problem can be formulated as follows:

Consider variable $X=\left[x_{1},x_{2},x_{3}\right]=\left[A_{s},d_{f},b\right]$;

minimize $F(X)=29.4x_{1}+0.6x_{2}x_{3}$;

subject to

$$g_{1}(X)=\frac{x_{2}}{x_{3}}-4\leq 0,$$

$$g_{2}(X)=180+7.375\frac{x_{1}^{2}}{x_{3}}-x_{1}x_{2}\leq 0;$$

variable range $x_{1}\in \left\{6,6.16,6.32,6.6,7,7.11,7.2,7.8,7.9,8,8.4\right\}$, $x_{2}\in \left\{28,29,\ldots ,40\right\}$, $5\leq x_{3}\leq 10$.

Table A7 shows four statistical indicators for RCO and other optimizers on this problem. Obviously, RCO gives results that surpass all algorithms on these indicators. It is worth mentioning that RCO and RFO can find the optimal solution *X* = (6.32, 34, 8.499999999999) that minimizes the cost in every run. In addition, DBO, HHO, GOA, DA, and MFO can also find the optimal solution through repeated attempts. From Figure A7, it can be noticed that the convergence behavior of RCO has a stepwise process and finally converges to the near-optimal solution of this problem.

**Table A7:** Evaluation results for reinforced concrete beam design problem optimized by RCO and other optimizers.

Optimizer	Mean	Std	Best	Worst
RCO	359.20799999	5.7815 × 10^−14^	359.20799999	359.20799999
DBO	360.01920035	1.3682 × 10^0^	359.20799999	362.24999999
GJO	359.21173823	3.6435 × 10^−3^	359.20801045	359.22462089
RUN	359.30940274	5.5539 × 10^−1^	359.20800001	362.25001392
SMA	359.32220003	6.2549 × 10^−1^	359.20800000	362.63400001
HHO	359.43832098	8.6872 × 10^−1^	359.20799999	362.63399999
SOA	359.23787686	2.6046 × 10^−2^	359.20928087	359.29587381
GOA	363.64311954	5.2455 × 10^0^	359.20799999	376.80000000
DA	359.61359999	1.0517 × 10^0^	359.20799999	362.24999999
MVO	359.21213089	4.5808 × 10^−3^	359.20805144	359.22277961
EGO	362.92917939	1.6793 × 10^0^	359.49427687	366.56462811
COA	362.16349552	2.9032 × 10^0^	359.20800093	373.47135412
ALO	359.65200005	1.1529 × 10^0^	359.20800000	362.63400012
MFO	360.70439999	1.6324 × 10^0^	359.20799999	362.63399999
RFO	359.20799999	6.5919 × 10^−14^	359.20799999	359.20799999
ABC	359.20855745	9.3011 × 10^−4^	359.20800023	359.21200306
